# Loss of Ciliary Gene *Bbs8* Results in Physiological Defects in the Retinal Pigment Epithelium

**DOI:** 10.3389/fcell.2021.607121

**Published:** 2021-02-18

**Authors:** Sandra Schneider, Rossella De Cegli, Jayapriya Nagarajan, Viola Kretschmer, Peter Andreas Matthiessen, Daniela Intartaglia, Nathan Hotaling, Marius Ueffing, Karsten Boldt, Ivan Conte, Helen Louise May-Simera

**Affiliations:** ^1^Faculty of Biology, Institute of Molecular Physiology, Johannes Gutenberg-University, Mainz, Germany; ^2^Telethon Institute of Genetics and Medicine, Pozzuoli, Italy; ^3^National Center for Advancing Translational Sciences, National Institutes of Health, Bethesda, MD, United States; ^4^Medical Bioanalytics, Institute for Ophthalmic Research, Eberhard-Karls University, Tübingen, Germany; ^5^Department of Biology, University of Naples Federico II, Naples, Italy

**Keywords:** cilia, ciliopathy, retinal pigment epithelium, genetic disease, molecular medicine, RPE, Epithelial-to-Mesenchym Transition (EMT)

## Abstract

Primary cilia are sensory organelles vital for developmental and physiological processes. Their dysfunction causes a range of phenotypes including retinopathies. Although primary cilia have been described in the retinal pigment epithelium (RPE), little is known about their contribution to biological processes within this tissue. Ciliary proteins are increasingly being identified in non-ciliary locations and might carry out additional functions, disruption of which possibly contributes to pathology. The RPE is essential for maintaining photoreceptor cells and visual function. We demonstrate that upon loss of *Bbs8*, predominantly thought to be a ciliary gene, the RPE shows changes in gene and protein expression initially involved in signaling pathways and developmental processes, and at a later time point RPE homeostasis and function. Differentially regulated molecules affecting the cytoskeleton and cellular adhesion, led to defective cellular polarization and morphology associated with a possible epithelial-to-mesenchymal transition (EMT)-like phenotype. Our data highlights the benefit of combinatorial “omics” approaches with *in vivo* data for investigating the function of ciliopathy proteins. It also emphasizes the importance of ciliary proteins in the RPE and their contribution to visual disorders, which must be considered when designing treatment strategies for retinal degeneration.

## Introduction

Primary cilia are microtubule-based sensory organelles extending from the cell membrane and are indispensable for a variety of developmental and physiological processes. As such, they are considered as signaling hubs that transmit extracellular signals, and are involved in regulating many signaling pathways, including Wnt, hedgehog, and transforming growth factor β (Tgf-β) (Fliegauf et al., 2007; Ishikawa and Marshall, 2011; May-Simera et al., 2017, 2018; Pala et al., 2017). Defects in primary cilia function or assembly lead to a wide range of diseases, collectively termed ciliopathies. Since nearly every cell exhibits a primary cilium, ciliary dysfunction leads to a multitude of different phenotypes, with retinopathy being the most common (Waters and Beales, 2011; May-Simera et al., 2017). The Bardet-Biedl syndrome (BBS) was one of the first ciliopathies described. *BBS* genes encode proteins required for ciliary trafficking, making them essential for maintenance and function of primary cilia and as such for development and homeostasis of various tissues and organs (Goetz and Anderson, 2010; Waters and Beales, 2011; Forsythe and Beales, 2013; Valverde et al., 2015). Ciliary proteins are increasingly being identified in non-ciliary locations and might carry out additional functions, disruption of which possibly contributes to pathology (Novas et al., 2015; Marchese et al., 2020).

Most research on the retinal aspect of ciliopathies has mainly focused on the photoreceptor, whose outer segment (POS) is a highly specialized primary cilium with the so-called connecting cilium akin to the ciliary transition zone. To date, there is limited information on the contribution of primary cilia in other ocular cell types. Probably, the most relevant ciliated tissue in addition to the photoreceptor cells is the retinal pigment epithelium (RPE). Our recent discoveries did indeed reveal an indispensable role for primary cilia in maturation and functional polarization of this tissue (May-Simera et al., 2018; Patnaik et al., 2019). The RPE is a monolayer of pigmented epithelial cells intercalated between the neural retina and the choriocapillaris of the eye and forms part of the blood-retinal barrier (BRB). With their long apical microvilli, RPE cells ensheath the light-sensitive POS leading to the functional interaction between both tissues (Strauss, 2005; Willoughby et al., 2010). The RPE has additional roles which are critically dependent on maintaining its epithelial phenotype: absorption of scattered photons, regeneration of 11*-cis* retinal in the visual cycle, phagocytosis of shed POS, secretion of various growth factors, transepithelial transport of nutrients and ions, and the maintenance of photoreceptor cells. Therefore, RPE dysfunction and associated failure of one or more of its functional processes are often linked with retinal degeneration and vision impairment (Strauss, 2005; Bharti et al., 2011; Chen et al., 2019).

We have previously shown that primary cilia are crucial for maturation and polarization of induced pluripotent stem cells (iPSC)-RPE *in vitro* (May-Simera et al., 2018). As a consequence of inefficient ciliogenesis and therefore of incomplete RPE maturation, the RPE cells showed reduced expression of adult RPE-specific genes, defective apical microvilli morphology, as well as reduced functionality. Confirming this, we demonstrated that primary cilia dysfunction *in vivo* leads to changes in RPE cell morphology, including underdeveloped tight junctions and apical microvilli in newborn (P0) cilia mutant mice (May-Simera et al., 2018). However, to date, long-term consequences of primary cilia dysfunction in the RPE remain to be investigated. The present study was aimed at elucidating these effects *in vivo* on the maturation and homeostasis of the RPE as the tissue ages. To do so, we again turned to the *Bardet-Biedl syndrome protein (Bbs)-*deficient mouse model, as *Bbs* knockouts display significantly reduced and dysfunctional primary cilia (Ross et al., 2005; Tadenev et al., 2011). The *Bbs8/Ttc8* gene encodes a component of the “BBSome,” a protein complex required for ciliary trafficking (Tadenev et al., 2011). We used a *Bbs8*-knock out model since loss of *Bbs8* has one of the most pronounced ciliopathy phenotypes particularly with regard to visual dysfunction (Tadenev et al., 2011; Dilan et al., 2018; May-Simera et al., 2018; Kretschmer et al., 2019; Patnaik et al., 2019).

Our data provides evidence of how loss of ciliary gene/protein *Bbs8* results in physiological defects affecting RPE homeostasis and function, characterized by alteration in both gene and protein expression profiles.

## Results

### Transcriptomic Analysis of *Bbs8-*Deficient RPE Reveals Mis-regulation of Genes Involved in Numerous RPE-Essential Processes

**Figure 1:**
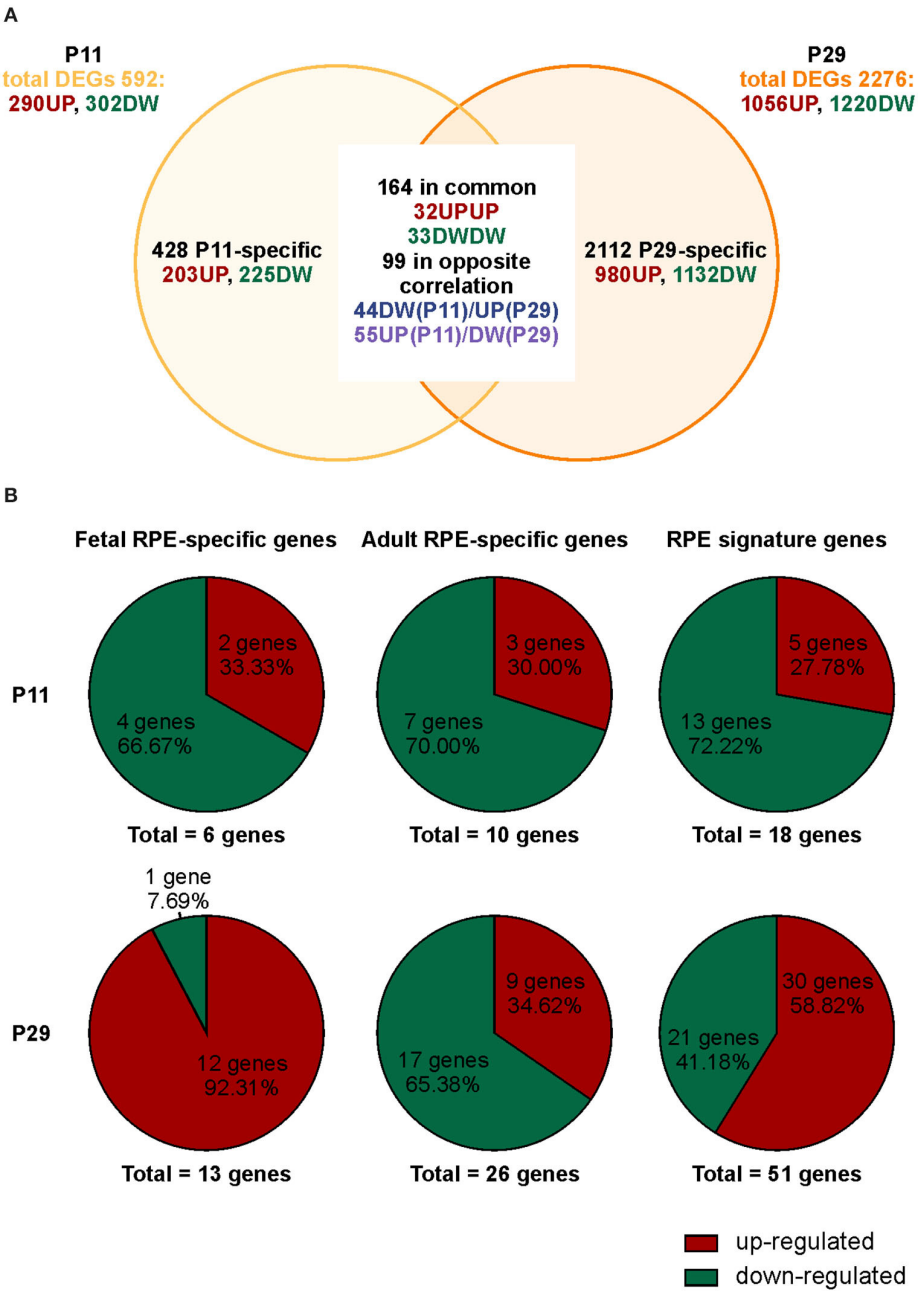
Loss of *Bbs8* induces a switch in RPE-specific gene expression from P11 to P29. **(A)** Venn diagram showing differentially expressed genes (DEGs) obtained via transcriptomic analysis. The number of specific and common DEGs and the orientation of expression are shown. DW, down-regulated; UP, up-regulated. **(B)** Pie charts depicting % up- or down-regulated genes for three different categories; fetal RPE-specific genes, adult RPE-specific genes and RPE signature genes. At P11 all categories have an increased down-regulation. At P29 fetal RPE-specific genes and RPE signature genes become up-regulated.

We previously demonstrated that *Bbs8*^−/−^ mice show incomplete maturation of the RPE at postnatal day 0 (P0) prior to development of the POS, due in part to over-activation of canonical Wnt signaling (May-Simera et al., 2018). However, whether and how ablation of *Bbs8* caused other developmental defects, including alterations of RPE maturation and its crosstalk with adjacent photoreceptor cells has been not been investigated. We first explored the phenotypic consequences caused by loss of *Bbs8* in the RPE by focusing on patterns of gene expression. We investigated the effect of *Bbs8* deletion in the RPE by carrying out an unbiased QuantSeq 3′ mRNA sequencing analysis of P11 and P29 RPE specimens isolated from *Bbs8*^−/−^ and *Bbs8*^+/+^ mouse eyes. At both time points, this analysis yielded differentially expressed transcripts in *Bbs8*^−/−^ RPE compared to littermate controls. At P11, we observed up-regulation of 290 transcripts and down-regulation of 302 transcripts (Figure 1A, Supplementary Tables 1, 3). At P29, 1,056 transcripts were up-regulated, and 1220 transcripts were down-regulated (Figure 1A, Supplementary Tabes 2, 4). This raised the possibility that loss of *Bbs8* may directly or indirectly influence expression of a large number of genes and alter RPE maturation and function. To better investigate this possibility, we compared the mis-regulated genes with lists of RPE-specific genes, divided into fetal RPE-specific, adult RPE-specific and RPE signature genes published in Strunnikova et al. (2010) (Figure 1B, Supplementary Table 5). Across all three categories a similar trend was observed at P11, namely that ~70% differentially expressed genes (DEGs) were down-regulated and ~30% were up-regulated (Fetal RPE-specific *n* = 6 genes, Adult RPE-specific *n* = 10 genes, RPE signature *n* = 18 genes). However, at P29 ~90% of the fetal RPE-specific genes were up-regulated, whereas ~65% of the adult RPE-specific genes were down-regulated (Fetal RPE-specific *n* = 13 genes, Adult RPE-specific *n* = 26 genes, RPE signature *n* = 51 genes). Suggesting a delay in RPE maturation in the mutants.

**Figure 2:**
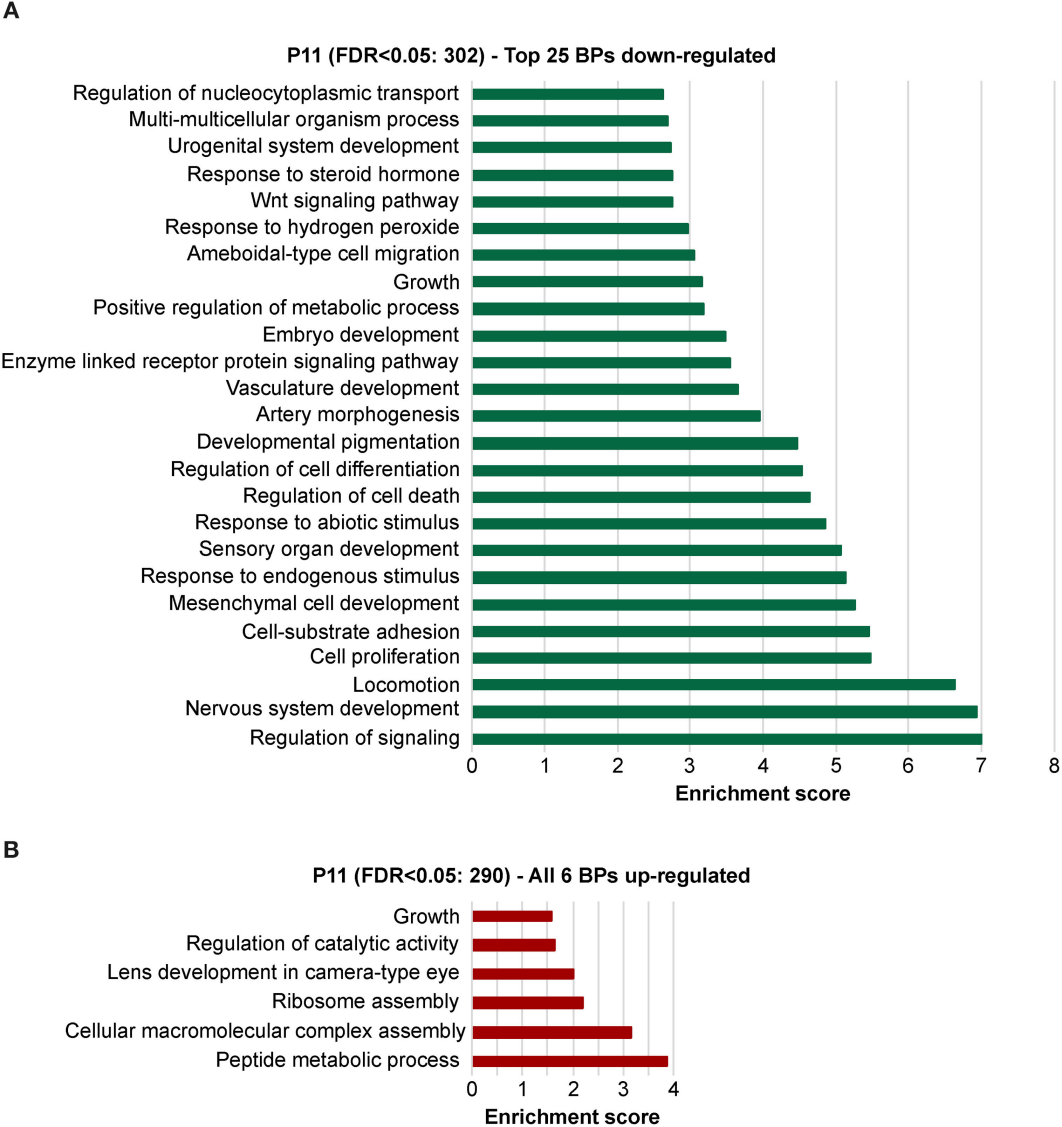
Identification of the biological processes underlying the effect of the deletion of *Bbs8* at P11 via gene ontology analysis. Top 25 significant Biological processes (BPs) among the inhibited transcripts **(A)** and the 6 induced **(B)** in *Bbs8*^−/−^ vs. *Bbs8*^+/+^. The Enrichment score for each BP cluster is plotted on the x-axis.

**Figure 3:**
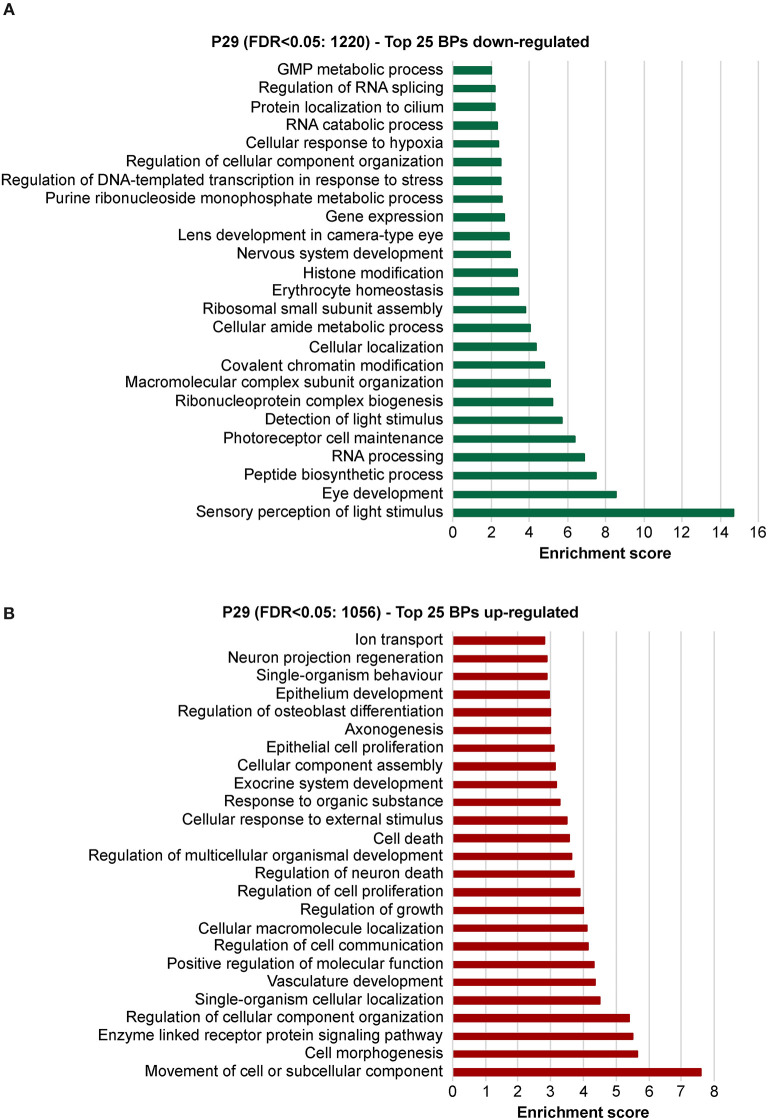
Identification of the biological processes underlying the effect of deletion of *Bbs8* at P29 via gene ontology analysis. Top 25 significant Biological pathways (BPs) among the inhibited transcripts **(A)** and the induced **(B)** in *Bbs8*^−/−^ vs. *Bbs8*^+/+^ are plotted. The Enrichment score for each BP cluster is plotted on the x-axis.

To explore the biological significances of these transcriptomes, we performed a gene ontology enrichment analysis (GOEA) restricting the output to biological process (BP) terms at each time point (see Figure 2 and Supplementary Table 6 for P11, and Figure 3 and Supplementary Table 7 for P29). Figure 2 shows the top 25 most significant down-regulated and all six up-regulated BP-clusters in which the inhibited and induced transcripts, respectively, were mainly functionally enriched. The Enrichment score (ES) represents the amount to which genes in a gene ontology cluster are over-represented. The ES scale can vary dependent on how genes cluster in a given term. At P11 most of the DEGs involved signaling and developmental processes (Figure 2, Supplementary Table 6), while at P29 they involved RPE homeostasis and function (Figure 3, Supplementary Table 7).

**Figure 4:**
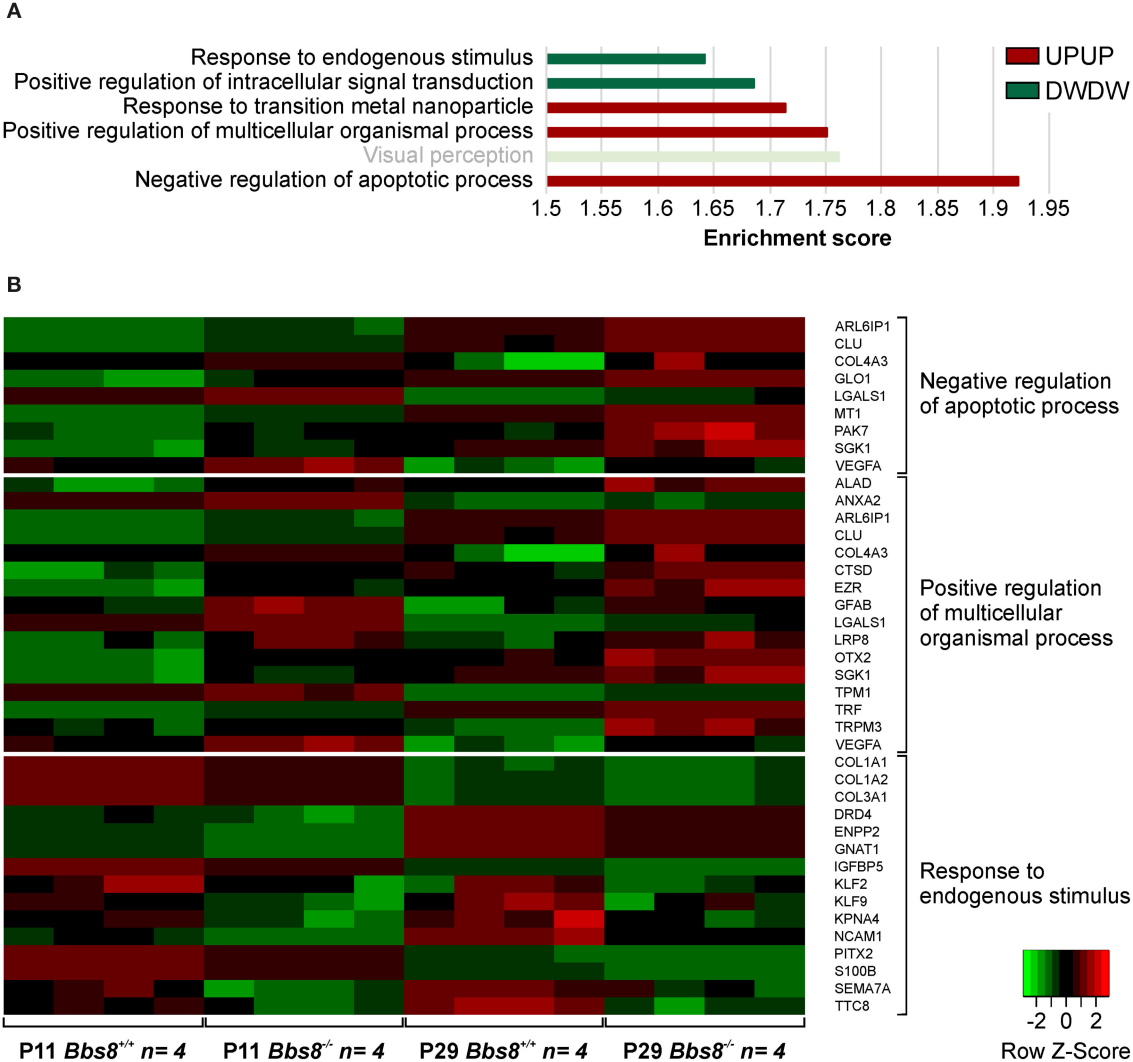
GOEA of commonly regulated DEGs from *Bbs8*^−/−^ RPE at P11 and P29. **(A)** We identified three clusters of Biological processes (BP) that were significantly up-regulated and three that were significantly down-regulated at both time points. Terms are ranked according to the Enrichment score of each BP cluster. Light bright green bar denotes photoreceptor-specific genes. DW, down-regulated; UP, up-regulated. **(B)** Heatmap of differentially regulated genes associated with processes in A.

A comparison of DEGs in *Bbs8*^−/−^ vs. control RPE at two timepoints, P11 and P29, identified 65 mis-regulated genes as shown in the Venn diagram in Figure 1A. Of these 32 were up-regulated and 33 down-regulated at both time points. We again performed GOEA analysis to identify common biological processes. We identified three clusters of biological processes that were significantly up-regulated and three that were significantly down-regulated. Up-regulated processes included responses to metal ions (albeit only four genes), processes involving cellular organization and apoptotic processes (Figure 4A, Supplementary Table 8). Down-regulated processes included responses to endogenous stimuli, processes involving signal transduction and visual perception (Figure 4A, Supplementary Table 8). Since the genes involved in visual perception included several photoreceptor-specific genes, we attributed this cluster to contamination of adjacent tissues (see discussion). The heatmap in Figure 4B visualizes differentially regulated genes associated with these processes. Many mis-regulated genes are involved in RPE polarization and function, receptors and channel proteins (*Trpm3, Drd4, Mt1*), cytokines (*Vegfa, S100B*) and genes encoding proteins involved in phagocytosis and cellular metabolism (*Anxa2, Ctsd, Ezr, Trf* ). Moreover, we detected an up-regulation of genes associated with apoptotic processes suggesting possible pathological changes in the *Bbs8* knockout RPE as early as P11, not necessarily just associated with a delay in maturation.

### Proteomic Analysis of *Bbs8-*Deficient RPE Suggests Loss of Functionality

**Figure 5:**
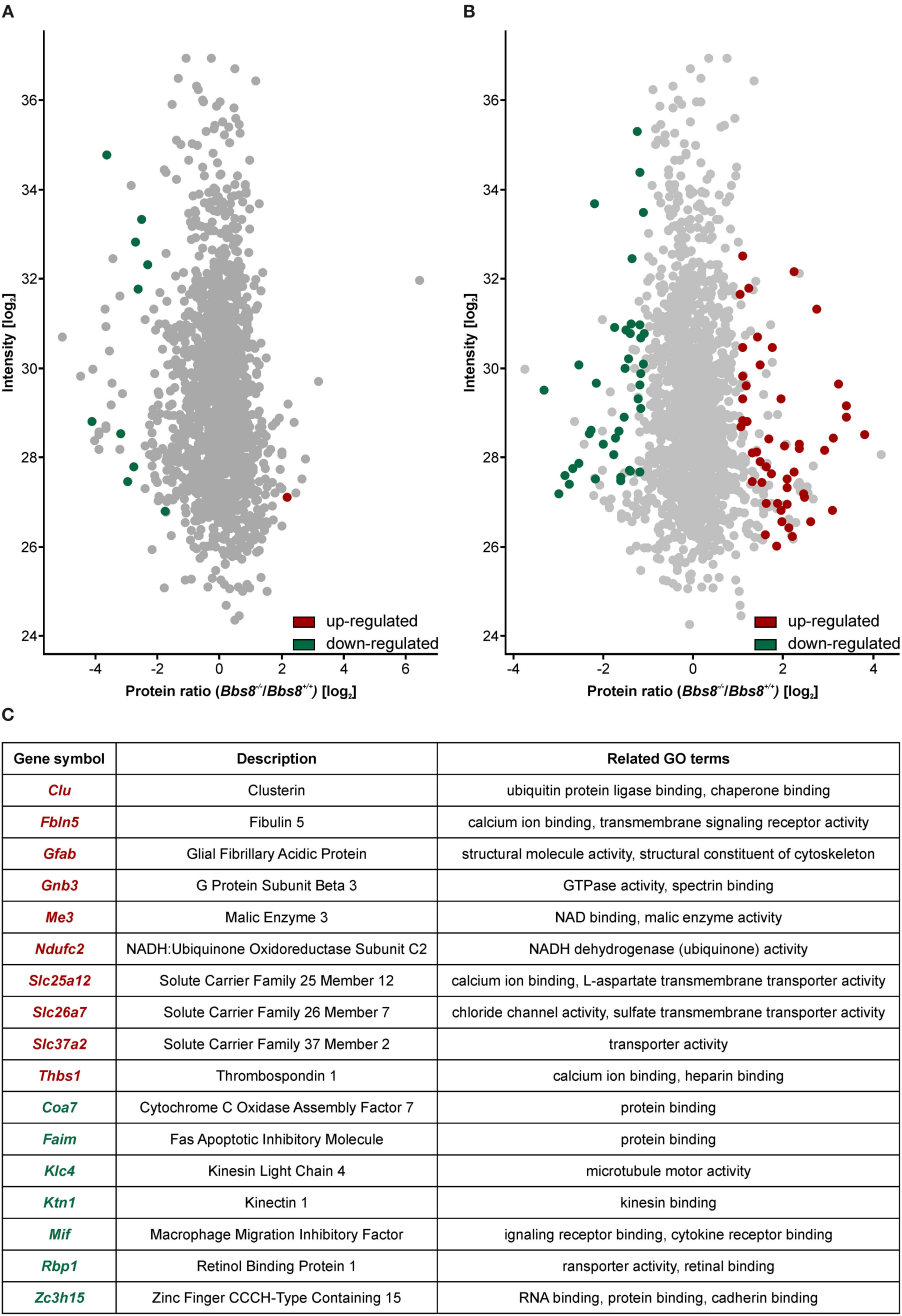
Proteomic analysis shows an increase in mis-regulated proteins in P29 *Bbs8*^−/−^ RPE. **(A)** Volcano plot showing significantly up-regulated (red) and down-regulated (green) proteins in P11 *Bbs8*^−/−^ RPE compared to controls. **(B)** Volcano plot showing significantly up-regulated (red) and down-regulated (green) proteins in P11 *Bbs8*^−/−^ RPE compared to controls. **(C)** Comparison of Proteome and Transcriptome data. Chart shows commonly up or down-regulated molecules at P29 with gene symbols, descriptions and related GO terms.

To better investigate the phenotypic consequences associated with Bbs8 depletion, we performed mass spectrometry-based quantitative proteomics. We analyzed RPE cells isolated from *Bbs8*^−/−^ and *Bbs8*^+/+^ mice at P11 and P29. At both time points, a significant mis-regulation of the proteome was observed. In agreement with the transcriptomic analysis, we observed more mis-regulation in P29 *Bbs8*^−/−^ RPE even at protein level. In *Bbs8*^−/−^ at P11 we detected ten proteins down-regulated and only one up-regulated (Figure 5A, Supplementary Table 9). Several of these down-regulated proteins were associated with photoreceptor outer segment (POS) processes. Since many of these proteins are not significantly changed at the transcript level it suggests that they might come from POS. Their down-regulation in the mutant might be due to alteration in POS processing in the RPE, in defects in the POS phagocytosis or both cellular processes. This last possibility is particularly attractive, because RPE cells begin to extend their microvilli by P5 to phagocytize shed POS around P11 (Mazzoni et al., 2014). Concordantly, our data also showed an increase in the protein expression of mitochondrial transcription factor A (Tfam). This data further indicated that Bbs8^−/−^ RPE showed a defect in the differentiation since, previous reports have highlighted the relevance of Tfam up-regulation in modulating cell differentiation (Collu-Marchese et al., 2015; Agostini et al., 2016).

At P29, 50 proteins were up-regulated and 40 down-regulated (Figure 5B, Supplementary Table 9). To gain insight into differentially regulated processes, we performed an enrichment analysis of the significantly dysregulated proteins. At P11 there were not enough molecules to perform enrichment analysis. At P29 we found integrin binding (GO:0005178) significantly mis-regulated, including adhesion proteins such as Paxillin, Thrombospondin, and Vitronectin. This data may suggest defects in cellular adhesion. In an attempt to consolidate our transcriptomic and proteomic datasets, we looked for overlapping molecules (transcripts/proteins). At P11 only one molecule (Gnat) was down-regulated in both data sets. At P29, ten molecules were up-regulated in both data sets whilst seven were down-regulated (Figure 5C). These include molecules involved in metabolism and cytoskeletal components. The proteomic data set at P29 contained at least eight other up-regulated proteins involved in metabolism (Atp5j, Cox5b, Slc25a11, Ndufa9, Timm9, Mtch1, Isca2, Timm8a1). It also contained other down-regulated cytoskeletal proteins associated with adhesion (Paxillin, Palmd, Limch1, Pdlim2, Ermn, Cttn). The mis-regulation of this subset of proteins is indicative of significant metabolic or physiologic changes likely affecting function and not just a delay in maturation in the RPE upon loss of Bbs8.

### Loss of *Bbs8* Results in Defective RPE-Cell Morphology

**Figure 6:**
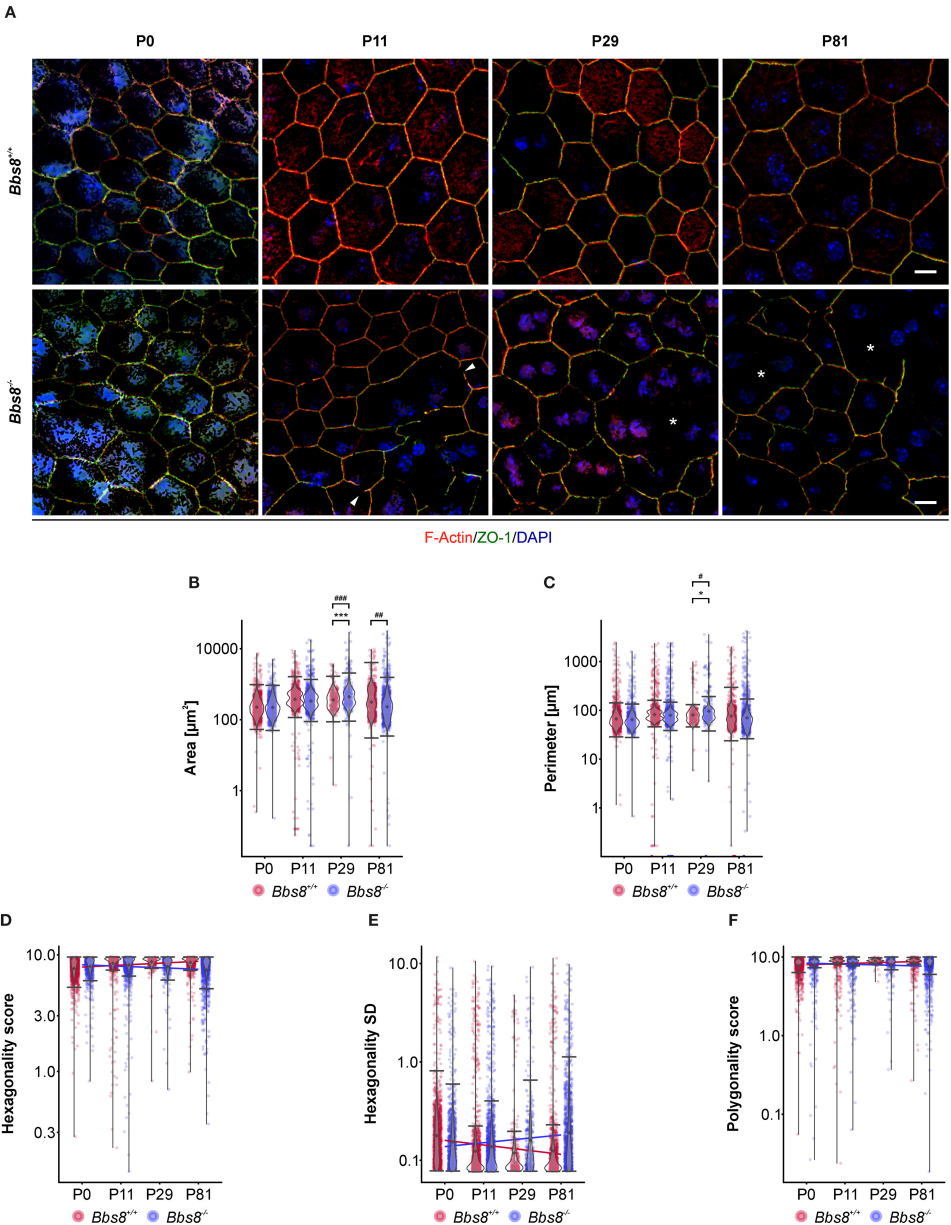
Cell morphological defects are seen in *Bbs8*-deficient RPE. **(A)** Representative images of RPE flatmounts stained for F-actin (red) and ZO-1 (green) to visualize cytoskeleton and cell membrane. DAPI was used to stain nuclear DNA. P11 *Bbs8*^−/−^ RPE shows discontinuous membrane staining (arrowheads). Starting in P29 *Bbs8*^−/−^ RPE shows larger areas of RPE cells with distorted or even completely disrupted cell membranes (asterisks). Scale bars: 10 μm. **(B–F)** Quantification of cell morphology parameters. Statistical analysis is described in methods. **(B)** Comparison of cell area of *Bbs8*^−/−^ and *Bbs8*^+/+^ RPE cells reveals an increase of the mean (*p* = 6.43 x 10^−45^) and the variance (*p* = 5.28 x 10^−52^) of P29 *Bbs8*^−/−^ RPE cells and an increase of variance (*p* = 0.0013) of P81 *Bbs8*^−/−^ RPE cells. **(C)** Comparison of cell perimeter of *Bbs8*^−/−^ and *Bbs8*^+/+^ RPE cells reveals an increase of the mean (*p* = 0.0342) and the variance (*p* = 0.0272) of P29 *Bbs8*^−/−^ RPE cells. **(D)** Comparison of hexagonality score of *Bbs8*^−/−^ and *Bbs8*^+/+^ RPE cells. The blue line depicts a trend toward cells being less hexagonal in the mutant over time. The opposite trend (red line) is observed in *Bbs8*^+/+^ RPE. **(E)** The standard deviation of the hexagonality score trends upwards in the mutant (blue line). The opposite trend is observed in *Bbs8*^+/+^ RPE (red line). **(F)** Comparison of polygonality score of *Bbs8*^−/−^ and *Bbs8*^+/+^ RPE cells reveals a trend to more polygonal cells in *Bbs8*^−/−^ RPE with higher age (blue line). The opposite trend is observed in *Bbs8*^+/+^ RPE (red line). (*n*: P0 *Bbs8*^+/+^ = 1,492 cells, P0 *Bbs8*^−/−^ = 1,030 cells, P11 *Bbs8*^+/+^ = 1,536 cells, P11 *Bbs8*^−/−^ = 1,755 cells, P29 *Bbs8*^+/+^ = 331 cells, P29 *Bbs8*^−/−^ = 383 cells, P81 *Bbs8*^+/+^ = 1,128 cells, P81 *Bbs8*^−/−^ = 1,315 cells) Significance levels: mean: > 0.05 not significant (ns), ≤0.05*, ≤0.01**, ≤0.001***; variance: > 0.05 not significant (ns), ≤0.05#, ≤0.01##, ≤0.001###.

Since several dys-regulated molecules were involved in adhesion, and RPE function is critically dependent on a tightly connected monolayer epithelium, we examined RPE cellular morphology and patterning in RPE flatmount preparations from *Bbs8*^−/−^ and *Bbs8*^+/+^ mice. We stained for F-actin, a cytoskeletal marker, and zonula occludens-1 (ZO-1), a tight junction protein, at different time points (P0, P11, P29, and P81) (Figure 6A). From P11 onwards, we observed a progressive loss of the typical hexagonal, honeycomb-like structure in *Bbs8*^−/−^ RPE cells compared to control. This morphological phenotype was accompanied by discontinuous staining of both F-actin and ZO-1 markers (arrowheads). Morphology of the RPE cells was significantly impaired and culminated in evident distortion or complete disruption of cytoplasmic membranes (asterisks) at P81. In contrast to this, cellular morphology in the control flatmounts remained consistent as the tissue aged (Figure 6A).

To investigate this further we assessed cellular morphology changes via high-content image analysis. Due to the nature of the disrupted morphology encountered in the mutant samples, damaged areas could not be included in the automatic software analysis (for an example see Supplementary Figure 1G). Epithelial parameters included area, perimeter, hexagonality, polygonality, eccentricity, and number of neighbors (Figures 6B–F, Supplementary Figures 1A–F). Even without the damaged regions, quantification of area revealed that *Bbs8*^−/−^ RPE cells were significantly larger compared to controls at P29, and the variance in cell area was significantly increased at P29 and at P81 (Figure 6B). P29 *Bbs8*^−/−^ RPE also showed a significant increase in cell perimeter and in the variance of cell perimeter (Figure 6C). Although not statistically significant, we observed that *Bbs8*^−/−^ RPE cells were becoming less hexagonal with age, which is opposite to normal *Bbs8*^+/+^ RPE (Figure 6D). A similar trend was observed with the Hexagonality SD (standard deviation) (Figure 6E). Although the majority of RPE cells lose their cilium as the tissue matures, these findings indicate that either ciliary function, even in just a small percentage of cells, is continuously required for maintenance of epithelial morphology. Alternatively, a subset of ciliary proteins (in particular the BBS proteins) may have additional alternative functions affecting cellular morphology.

### Loss of *Bbs8* Leads to Functional RPE Defects

**Figure 7:**
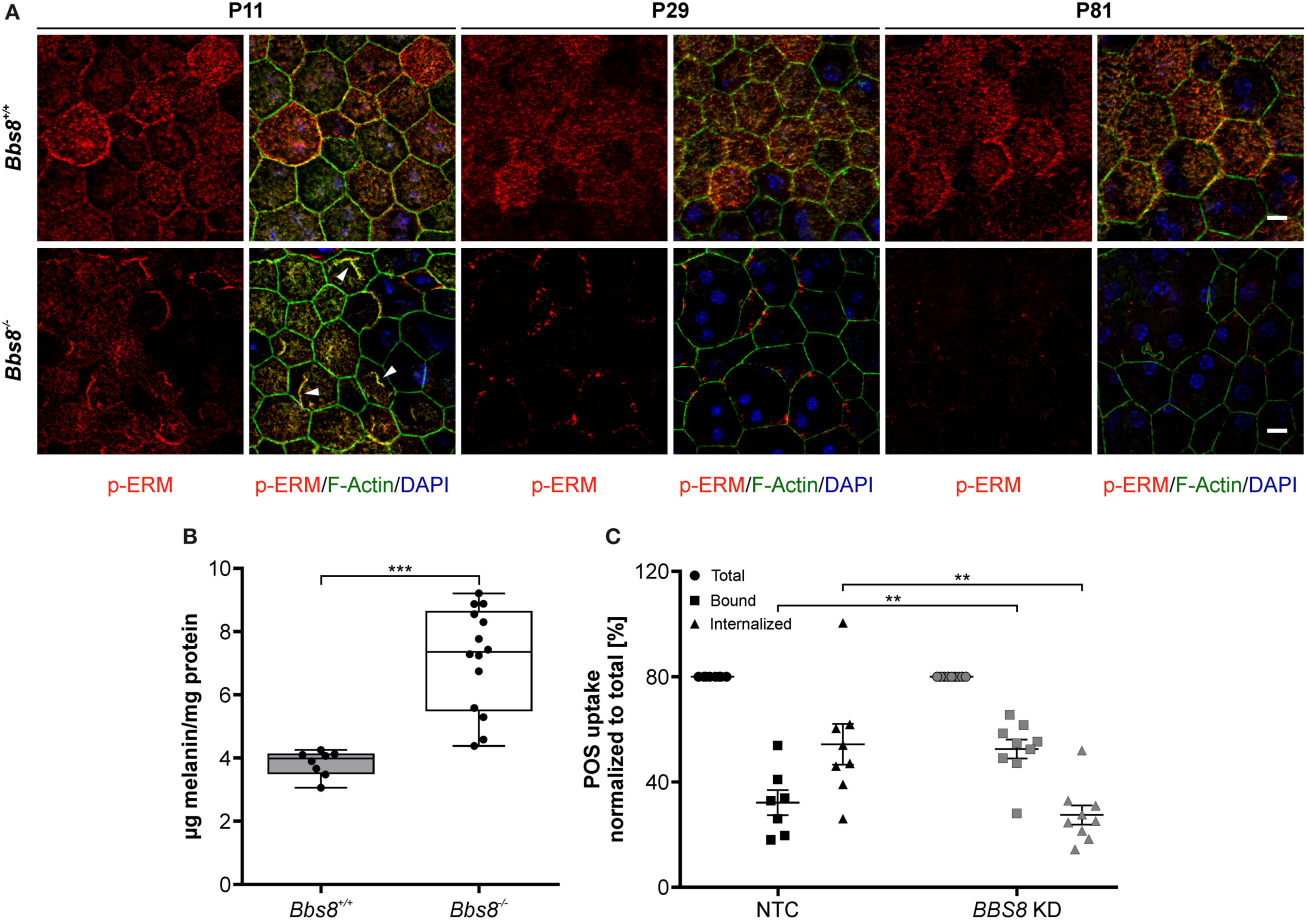
Deletion of *Bbs8* leads to defects in RPE function**. (A)** Representative images of RPE flatmounts stained for p-ERM (red) and F-actin (green) to visualize apical microvilli and the cytoskeleton. DAPI was used to stain nuclear DNA. P11 *Bbs8*^−/−^ RPE shows abnormal accumulations of p-ERM staining (arrowheads). Staining of p-ERM is dramatically reduced as the tissue ages. *Bbs8*^+/+^ RPE shows consistent staining at all ages. Scale bars: 10 μm. **(B)** Retinal adhesion assay. Quantification of melanin attached to the retina is significantly increased in P16 *Bbs8*^−/−^ compared to controls (*p* < 0.0001). Statistical analysis was performed using ROUT test (Q = 0.1 %) identified 2 outliers before using unpaired *t*-test (*n*: *Bbs8*^−/−^ = 14, *Bbs8*^+/+^ = 8). **(C)** Phagocytosis assay. Quantification of photoreceptor outer segments (POS) uptake reveals a significant increase in bound POS (*p* = 0.0025) and a significant down-regulation in internalized POS (*p* = 0.0025) in *BBS8 KD* ARPE-19 cells compared to controls. Statistical analysis was performed using ROUT test (Q = 10%) identified 1 outliers before doing a one-way ANOVA and Tukey *post-hoc* test (*n*: *BBS8 KD* = 9, *NTC* = 7–8). Significance levels: ≤0.01**, ≤0.001***.

Disrupted morphology can consequently lead to a loss of polarization, which is essential for RPE functionality. In the RPE, apical microvilli are intricately connected to the adjacent POS and are indispensable for retinal homeostasis. Ezrin, a member of the Ezrin/Radixin/Moesin (ERM) family, is a key marker for microvilli, found in epithelial microvilli, where it bridges the actin cytoskeletal filaments and the cell membrane (Bonilha et al., 2006; Ohana et al., 2015). With *Bbs8* deletion *Ezrin* transcripts were significantly up-regulated, in contrast to down-regulation at the proteomic level at both ages. This might suggest a possible compensatory regulatory effect due to dysfunctional protein, in which the absence of a functional protein stimulates the cell to increase transcription (negative feedback). We stained *Bbs8-*deficient RPE flatmounts with anti-phospho-Ezrin(Thr567)/Radixin(Thr564)/Moesin(Thr558) (p-ERM), which marks functional Ezrin at apical microvilli (Ohana et al., 2015). In P11 *Bbs8*^−/−^ RPE, we observed decreased p-ERM staining compared to control, although occasionally there was irregular accumulation at the apical surface (Figure 7A arrowheads, Supplementary Figure 2 arrowheads). At a later time point (P81) p-ERM was predominantly absent (Figure 7A) which suggests a worsening of apical microvilli formation in *Bbs8-*deficient RPE as already described at P0 (May-Simera et al., 2018).

Defective apical microvilli undoubtedly have a consequent effect on the functional connection between RPE and POS. To verify this, we assessed the retinal adhesion between RPE and adjacent retina at P16. We quantified the amount of melanin in RPE apical microvilli still attached to the retina after it is mechanically separated from the RPE. Compared to controls, *Bbs8*^−/−^ retina showed higher concentration of melanin, this could mean that despite apical microvilli abnormalities, retinal adhesion was stronger or that there was defective directional movement of melanosomes back to the cell body (Figure 7B). To further investigate RPE function upon loss of *Bbs8*, we performed a phagocytosis assay on *BBS8 KD* ARPE-19 cells using siRNA. Due to the volume of material required and difficulty in obtaining *Bbs8*^−/−^ material, it was not possible to perform this assay in primary RPE cultures. We adapted a protocol previously described by Nandrot et al. (2007). *BBS8* knockdown was optimized and validated using qPCR (Supplementary Figure 3A) and assay conditions were tested prior to performing the experiments (Supplementary Figure 3B). *BBS8 KD* ARPE-19 cells showed significant up-regulation of bound POS compared to control, although the internalization, or more specifically the phagocytosis, of POS was significantly decreased in *BBS8 KD* cells (Figure 7C). Combined this data shows that loss of *Bbs8* disrupts processes involving functional apical processes.

### Loss of *Bbs8* Induces EMT-Like Traits in the RPE

**Figure 8:**
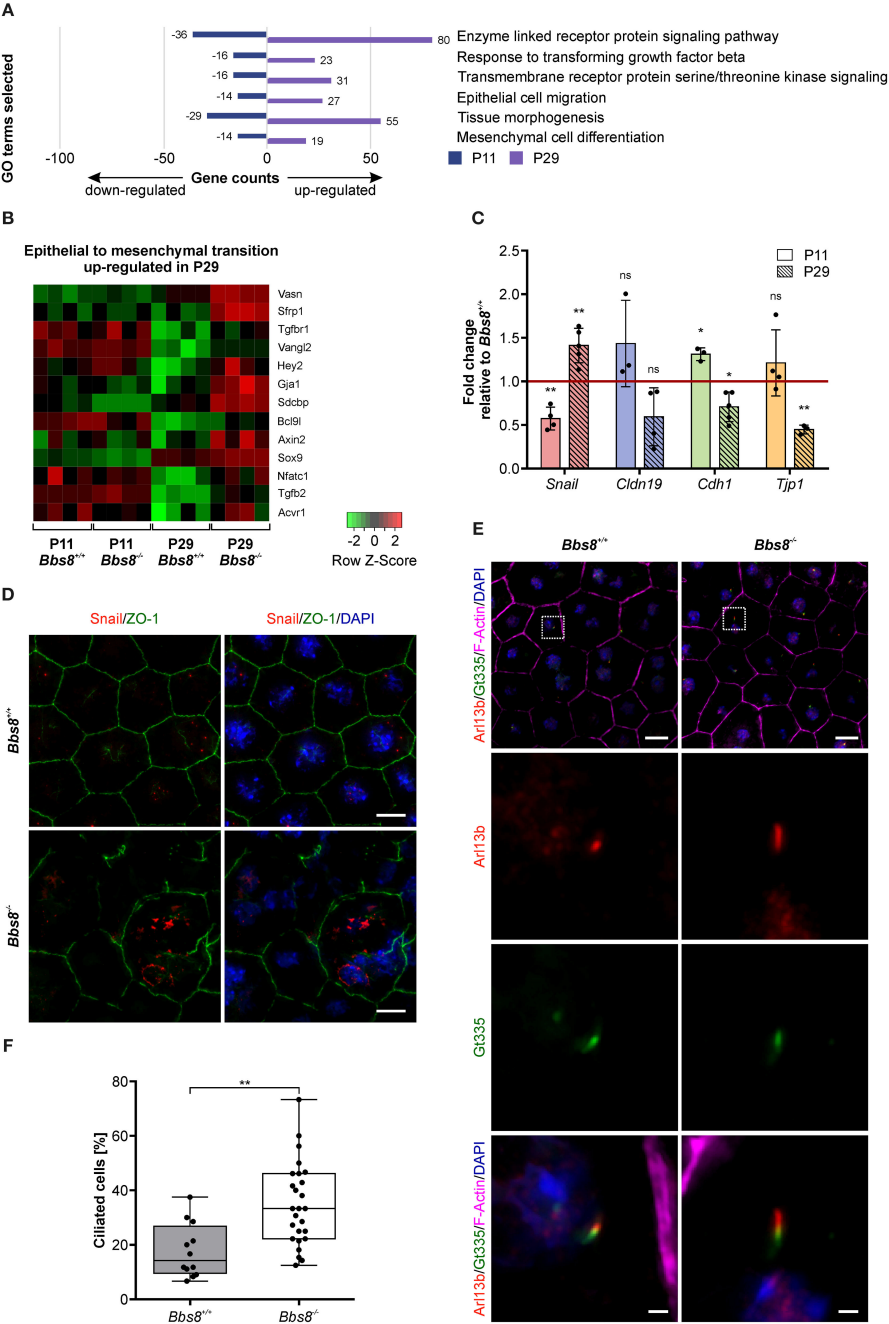
Loss of *Bbs8* induces EMT- like traits in the RPE. **(A)** GO terms associated with differentially expressed genes that were initially down-regulated at P11 and then up-regulated at P29. **(B)** Heatmap showing DEGs associated with epithelial-to-mesenchymal transition in the four experimental groups. *shi* **(C)** Quantitative gene expression shows a shift toward EMT-like associated gene expression profiles between P11 and P29. *Snail* expression shifts from down-regulation at P11 (*p* = 0.0073) toward up-regulation at P29 (*p* = 0.0096). Expression of *Cldn19, Cdh1*, and *Tjp1* shifts from an up-regulation (*Cldn19: p* = 0.2681*, Cdh1: p* = 0.0180 and *Tjp1: p* = 0.3439) at P11 toward a down-regulation at P29 (*Cldn19: p* = 0.0919*, Cdh1: p* = 0.0189 and *Tjp1: p* = 0.0028) (P11 n: *Bbs8*^−/−^ = 3–4, *Bbs8*^+/+^ = 4; P29 *n*: *Bbs8*^−/−^ = 3–5, *Bbs8*^+/+^ = 3–5). Statistical analysis was performed using one sample *t*-test. *Cdh1: E-cadherin, Cldn19: Claudin 19, Snail: Snail, Tjp1: tight-junction protein 1*. **(D)** Representative images of P11 RPE flatmounts stained for Snail (red) and ZO-1 (green). DAPI was used to stain nuclear DNA. P11 *Bbs8*^−/−^ RPE shows an increase in Snail expression especially in cells with disrupted morphology. Scale bars: 10 μm. **(E)** Representative images of RPE flatmounts at P29, stained for primary cilia using Arl13b (red) and Gt335 (green). F-Actin (magenta) was stained to visualize the cytoskeleton and DAPI was used to stain nuclear DNA. Higher magnifications are indicated in the image by a square. Scale bars: 10 μm, higher magnifications 1 μm. **(F)** Quantification of ciliated cells in P29 *Bbs8* RPE showing a significant increase in ciliation in *Bbs8*-deficient RPE compared to control (*p* = 0.0011) (*n*: *Bbs8*^−/−^ = 4 eyes, 340 cells, *Bbs8*^+/+^ = 2 eyes, 157 cells). Statistical analysis was performed using unpaired *t*-test. Significance levels: > 0.05 not significant (ns), ≤ 0.05*, ≤ 0.01**, ≤ 0.001***.

Previous studies have shown that ciliary mutations induce epithelial-to-mesenchymal transition (EMT) in other organs and tissues, including epicardial tissue, kidney epithelial cells, and pancreatic β-cells (Guen et al., 2017; Blom and Feng, 2018; Han et al., 2018; Volta et al., 2019). EMT denotes the trans-differentiation of epithelial cells into mesenchymal cells and is manifested by loss of cell junctions and apical-basal polarity, as well as reorganization of the cytoskeleton and changes in signaling and gene expression associated with cell shape (Lamouille et al., 2014). Since our omics data showed differential expression of molecules involved in adhesion and polarization and our patterning data identified changes in cell morphology, we looked specifically for EMT-related changes upon loss of *Bbs8*. Virtually all GO terms associated with differentially expressed genes that were initially down-regulated at P11 and then up-regulated at P29 were associated with EMT-related processes (Figure 8A). Similarly, a heatmap visualizing genes involved specifically in EMT revealed an up-regulation at P29, although not at P11 (Figure 8B). To support our transcriptomic data, we performed qPCR of EMT hallmark genes in *Bbs8*^−/−^ eyecups. Changes in the gene expression of the EMT transcription factor Snail have previously been detected in RPE cells undergoing EMT (Tamiya and Kaplan, 2016). Increased Snail is known to repress expression of E-cadherin and claudins (Lamouille et al., 2014). Furthermore, ZO-1 expression has been shown to be reduced upon EMT (Lamouille et al., 2014). In agreement with these observations, our qPCR data showed a shift toward an EMT-like state between P11 and P29 (Figure 8C). At P11 we saw a significant down-regulation of *Snail*, which accompanied a trend toward up-regulation of *Cldn19* and *Tjp1* and a significant induction of *Cdh1* compared to controls. In contrast, at P29 we detected a significant induction of *Snail* correlated to the significant down-regulation of *Cdh1* and *Tjp1*, together with a trend toward a down-regulation of *Cldn19* compared to controls. These opposite effects of EMT hallmark gene expression between P11 and P29 further supports the possibility that these alterations are mediated by the development of EMT-like characteristics over time. This finding is consistent with the observed progression of disrupted RPE cell morphology (Figure 6). In further demonstration that disrupted regions in the RPE could be linked to EMT, we stained P11 RPE flatmounts for Snail and observed an increase in Snail expression in mutant cells with disrupted morphology (Figure 8D, Supplementary Figure 4A). Snail expression was mostly localized to the nucleus in damaged areas and was not detected in control tissue.

EMT denotes the trans-differentiation from an epithelial to a mesenchymal cell (Ohlmann et al., 2016). This process includes de-differentiation, as well as *de novo* differentiation of cells of an epithelial cell layer. EMT has been linked to primary ciliogenesis as a mechanism of EMT programs (Guen et al., 2017) therefore, we examined ciliation in our mutant tissue. Primary cilia were identified via co-localization of ciliary membrane marker Arl13b and transition zone marker Gt335. To denote a true primary cilium, Arl13b staining must extend beyond Gt335 staining (Figure 8E). We had previously shown that the percentage of ciliated cells in mouse RPE is transient and reduces to under 20% in mature RPE. Similarly, we found that at P29 around 15% of control RPE cells were ciliated. In *Bbs8*^−/−^ RPE we observed a significant increase with almost twice as many ciliated cells (Figure 8F). In *Bbs8*^−/−^ RPE we also observed cilia at P81, whereas these were never detected in controls (Supplementary Figure 4B). This increase in ciliation in *Bbs8*^−/−^ RPE might be consistent with a failure of ciliary disassembly since we had previously seen a similar level of ciliation in in *Bbs8*^−/−^ RPE at E16.5, and could contribute to the development of a possible EMT-like phenotype observed at P29.

## Discussion

Ciliary mutations lead to a range of pathological phenotypes with retinal dystrophy being one of the most common. Not only do they cause syndromic retinal disorders, but they also underlie numerous non-syndromic retinal dystrophies and a large proportion of genes associated with vision loss directly encode ciliary proteins (https://sph.uth.edu/Retnet/). So far, most research on these retinopathies has focused on the highly specialized primary cilium of the photoreceptors, which undoubtedly plays a significant role in disease progression. However, there is little information available on the contribution of dysfunctional ciliary proteins in other ocular cell types (May-Simera et al., 2017, 2018). The RPE is a ciliated epithelial monolayer intercalated between the choriocapillaris and the retina and is indispensable for photoreceptor health and function (Strauss, 2005; May-Simera et al., 2018). Due to the close interaction between the RPE and the photoreceptors of the retina, they are often considered as a functional unit. We recently showed that primary cilia in the RPE are required for its maturation (May-Simera et al., 2018; Patnaik et al., 2019). Here we demonstrate that, upon ciliary disruption via deletion of *Bbs8*, the RPE never fully matures and exhibits phenotypic defects that affect RPE function even before adjacent photoreceptors have differentiated. This developmental defect is supported by a number of observations. Deletion of *Bbs8* leads to changes in gene and protein expression not only involving signaling pathways and developmental processes, but also at a later time point RPE homeostasis and function. Differentially regulated molecules affecting the cytoskeleton and cellular adhesion led to defective cellular polarization and morphology consequently disrupting phagocytic functions. Our combinatorial “omics” approach (QuantSeq 3′ mRNA sequencing and Mass spectrometry) strongly supported our *in vivo* data and highlights the value of multi-omic approaches in identifying *in vivo* molecular mechanisms in the RPE.

Importantly, as RPE and photoreceptor cells are in close physical contact to each other, we noticed contamination of photoreceptor tissue. The latter may arise from an alteration of extracellular matrix between photoreceptor and RPE and/or precocious degeneration events in the outer segments of Bbs8^−/−^ photoreceptors. In particular, at P29 we found changes involved in sensory perception of light stimulus in the RPE in the Bbs8^−/−^ mouse. Although this might be attributable to contamination events, it is also in accordance with recent data published by Liu et al. (2019), which highlighted the capacity of not completely differentiated RPE to produce essential visual cycle genes (Liu et al., 2019). Interestingly, in the proteomic data at P11 we found several photoreceptor outer segment (POS) proteins to be down-regulated compared to control. Since most of these are not significantly changed at the transcript level, it suggests that these do indeed come from POS in the control. This might be due to alteration in POS processing in the RPE or due to defects in the POS phagocytosis. It could also suggest a technical difference in that the preparations from control animals had closer attachment to the outer segments, thereby leaving more contamination upon separation. This in turn could represent a defect in the OS, but also a loss of attachment between the RPE and OS in the mutant animals.

Extrapolating from these, it would suggest that the P11 transcript changes are mostly RPE specific and strongly support an alteration in RPE differentiation and function. Furthermore, the availability of comparable gene expression level for a large number of RPE-specific genes rendered our results robust with regard to contaminant variation. Moreover, the majority of mis-regulated genes were exclusively involved in RPE polarization and function, rather than to a generic RPE gene expression changes.

An important point to consider is that our mouse model is not an RPE conditional knockout, meaning that adjacent photoreceptors also lacked *Bbs8*. We and others have already shown that loss of *Bbs8* results in early onset retinal degeneration with significant photoreceptor outer segment disruption (Dilan et al., 2018; Kretschmer et al., 2019). Although substantial photoreceptor outer segment defects can cause a secondary effect in the RPE, photoreceptor-dependent degeneration does not necessarily go hand in hand with early RPE cell loss or gross RPE morphology. This can be seen in other mouse models with photoreceptor specific mutations (such as peripherin and rhodopsin), which do not have an early RPE phenotype despite retinal degeneration (Cheng et al., 1997; Liu et al., 2010). Since we observed changes in the RPE as early as P11, a time point at which the photoreceptor outer segments are still forming, we hypothesize that loss of Bbs8 in the RPE intrinsically leads to abnormalities, which likely become amplified when interacting with defective photoreceptors. Ideally this could be answered by generating RPE-specific *Bbs8* mutant mice. However, since retinopathy patients inevitably have mutations affecting all ocular tissues, there is still value in understanding the mechanisms of disease progression in global mouse mutants.

Bbs8 is an integral component of the BBSome required for ciliary trafficking, and countless studies have shown that loss of Bbs8 compromises ciliary function (Ansley et al., 2003; Blacque et al., 2004; Tadenev et al., 2011; Hernandez-Hernandez et al., 2013; May-Simera et al., 2015; Goyal et al., 2016). As primary cilia are essential for early developmental and physiological processes, their dysfunction may have long-term effects on the RPE (Fliegauf et al., 2007; Ishikawa and Marshall, 2011; May-Simera et al., 2017, 2018; Pala et al., 2017). However, the percentage of ciliated cells in the RPE varies over time from over 70% between embryonic day (E) 14.5 and E16.5 to around 10% after birth and in adult (May-Simera et al., 2018; Patnaik et al., 2019). If we consider Bbs8 function to be restricted to ciliary processes, then this either means that the changes we describe in this study are a consequence of earlier ciliary dysfunction, or that residual ciliated cells in some way contribute to RPE homeostasis. In this case, the increase in ciliation in *Bbs8* mutant RPE at P29 and beyond might be contributing to the phenotype. Alternatively, Bbs8 might also exert other functional roles, not directly associated with the primary cilium.

Additional roles for “traditional” ciliary proteins have recently been identified for numerous classes of ciliary proteins, in particularly the BBS proteins (Novas et al., 2015; Marchese et al., 2020). These might include membrane trafficking, cytoskeletal organization, or transcriptional regulation (Gascue et al., 2012; Hernandez-Hernandez et al., 2013; Patnaik et al., 2020). Interestingly, the transcriptomic data at P29 identified several biological processes associated with the nucleus, such as many RNA-related processes, transcription, histone/chromatin modification, and gene expression. Addressing this question will require the generation and comparison of various different cilia/Bbs-specific conditional mutant mice, as well as a more detailed elucidation of Bbs function in epithelial tissues.

Although Bbs8 has the potential to regulate multiple cellular processes, our omics data identified specific processes associated with a change in RPE phenotype. At P11 we identified mis-regulation of genes/proteins particularly associated with signaling and developmental processes, while at P29, we observed mis-regulation of gene/protein expression associated with functional processes. These changes were reflected upon assessment of cellular morphology *in vivo*, in that we observed a loss of cellular epithelial morphology, as well as progressive disruption of cell membranes. Since RPE function is critically dependent on the maintenance of its epithelial phenotype, it was not surprising to find loss of RPE functionality upon loss of ciliary function (Strauss, 2005; Bharti et al., 2011; Adijanto et al., 2012; Chen et al., 2019). Maintenance of an epithelial phenotype is crucial for apical-basal polarization of the tissue. This facilitates ezrin-rich apical microvilli to extend from the apical surface and engulf the POS. These microvilli (also referred to as apical processes) are required for the phagocytosis of shed POS and the high level of cross talk between RPE and photoreceptor cells. Loss of apical microvilli upon depletion of *Ezrin* causes photoreceptor degeneration *in vivo* (Bonilha et al., 2006). We saw that loss of *Bbs8* leads to abnormal accumulation of p-ERM early on, with almost complete absence at a later time point (P81). This observation suggests a loss of polarization or inability to form apical microvilli. Previous reports have proposed that apical microvilli are indispensable for the functional connection between RPE cells and the POS, yet defects in apical microvilli undoubtedly lead to RPE dysfunction (Bonilha et al., 2006; Nandrot et al., 2007). In line with this, we observed that *Bbs8*^−/−^ RPE displayed an increase in retinal adhesion *in vivo*, and knockdown of *Bbs8* resulted in decreased phagocytosis of POS *in vitro*.

EMT denotes the trans-differentiation from epithelial cells into mesenchymal cells and loss of an epithelial phenotype. Key processes in this include changes in signaling, reorganization of the cytoskeleton, as well as loss of cell junctions and apical-basal polarity (Lamouille et al., 2014; Ohlmann et al., 2016). In our data we found three GO terms (mesenchymal cell differentiation, epithelial cell migration, and membrane organization) associated with these processes, that were significantly down-regulated at P11, but significantly up-regulated by P29. This, coupled with the recent findings that dys-regulation of primary cilia signaling has already been shown to induce EMT in other tissues and organs, including epicardial tissue, basal mammary stem cells, and kidney epithelial cells (Lamouille et al., 2014; Ohlmann et al., 2016; Guen et al., 2017; Blom and Feng, 2018; Han et al., 2018), makes us believe that a loss of *Bbs8* might induce an EMT-like phenotype. In support of this the downstream EMT-transcription factor Snail was only found in the nuclei of *Bbs8* mutant tissue.

RPE dysfunction can lead to retinal degeneration and blindness (Strauss, 2005; Bharti et al., 2011; Adijanto et al., 2012; Chen et al., 2019). So far, most research on retinal degeneration in ciliopathy patients has targeted photoreceptors. Our findings highlight that the contribution of defective cilia or ciliary proteins may be more complex than initially envisaged, and that their function in the biological processes within in the RPE must also be taken into consideration. This is relevant for syndromic ciliopathy patients and also for others affected by non-syndromic retinal degeneration as a consequence of mutations in a ciliary disease gene. These considerations warrant attention when designing treatment strategies for retinal degeneration.

## Materials and Methods

### Animals

All experiments had ethical approval from the Landesuntersuchungsamt Rheinland-Pfalz and were performed in accordance with the guidelines given by the ARVO Statement for the Use of Animals in Ophthalmic and Vision Research. Animal maintenance and handling were performed in line with the Federation for Laboratory Animal Science Associations (FELASA) recommendations. Animals were housed in a 12-h (h) light/dark cycle. The morning after mating was considered as embryonic day (E) 0.5 and up to 24 h after birth was considered as postnatal day (P) 0. Animals were sacrificed by cervical dislocation. Generation of *Bbs8*^−/−^ mice and genotyping was previously described (Tadenev et al., 2011). Tissues and samples were collected from two groups, namely age-matched control (*Bbs8*^+/+^) and mutant (*Bbs8*^−/−^) littermates. Each individual animal was considered a biological replicate. A minimum of three biological replicates were used for each experiment. For RNA sequencing a minimum of four biological replicates was used.

### Antibodies

For immunofluorescence, the following primary antibodies were used: anti-phospho-Ezrin (Thr567)/Radixin(Thr564)/Moesin(Thr558) (p-ERM) (rb, 1:100, Cell Signaling Technology, #3141), anti-snail (rb, 1:100, Cell Signaling Technology, #3879), anti-Arl13b (rb, 1:800, Proteintech, #17711-1-AP), and anti-Gt335 (mM, 1:800, Adipogen, #AG-20B-0020). These antibodies were detected using the appropriate Alexa Fluor (AF)-488,−555, and−647 (1:400; Molecular Probes) conjugated secondary antibodies. Anti-Zonula Occludens-1 (ZO-1) was directly conjugated with AF-488 (1:100, ZO-1-1A12, Invitrogen, 339188) and Phalloidin was directly conjugated with AF-647 (1:40, Cell Signaling Technology, #8940).

### Fluorescence Staining and RPE Flatmount Preparation

Mice were sacrificed, eyes were enucleated and adjacent tissues, such as muscle and fat, cornea, lens, and retina were removed. Remaining eyecups were fixed with 4% paraformaldehyde (PFA) in 1 × phosphate-buffered saline (PBS) for 1 h. Following fixation, the eyecups were washed three times with 1 × PBS, incubated with 50 mM NH_4_Cl for 10 min and permeabilized with 1 × PBS 0.1% Tween-20 (PBST) with 0.3% Triton-X (TX) (PBST-TX) for 1 h and blocked with blocking buffer (0.1% ovalbumin, 0.5% fish gelatin in 1 × PBS) for 1 h. Following this, eyecups were incubated with primary antibodies over night at 4°C. Eyecups were washed three times with 1 × PBS and incubated with secondary antibodies, directly conjugated antibodies, and DAPI (Carl Roth) for 2 h in the dark. Post staining, two washing steps with PBST-TX and one with 1 × PBS for 20 min were performed, followed by mounting on microscope slides using Fluoromount-G (SouthernBiotech). Flatmounts were imaged using a Leica DM6000B microscope. Deconvolution (BlindDeblur Algorithm, one iteration step) and maximum projection were performed using Leica imaging software (Leica, Bensheim, Germany). Images were processed via Fiji using color correction and contrast adjustment (Schindelin et al., 2012).

### RPE Cell Isolation

For RPE cell isolation, we adapted a protocol previously described (Nandrot et al., 2004). Mice were sacrificed, eyes were enucleated and placed in ice-cold 1 × Hank's balanced salt solution (Ca^2+^-Mg^2+^-free) (HBSS-) (Gibco, #14175-095) with 0.01 M Hepes (Gibco, #15630-080) (HBSS-H-). Adjacent tissues, such as muscle and fat, cornea, lens, and retina were removed and eyecups were placed in 1.5 ml trypsin (2 mg/ml) (Difco trypsin 250, BD #215240) in 1 × Hank's balanced salt solution (with Ca^2+^ and Mg^2+^) (HBSS+) (Gibco, #14025-092) with 0.01 M Hepes (HBSS+)-H and incubated for 5 min (P11) or 30 min (adult) respectively at 37°C. Following this, the eyecups were transferred into ice-cold (HBSS+)-H and RPE sheets were peeled off the choroid. In case of P11, eyecups were then again incubated in trypsin for 2 min in 37°C and remaining RPE sheets were peeled off. RPE sheets were transferred to a microfuge tube and washed three times with ice-cold (HBSS+)-H (9391 rcf, 1.5 min, 4°C). RPE cells were then pelleted (9391 rcf, 1.5 min, 4°C), supernatant was removed and the RPE pellet was snap-frozen in liquid nitrogen. RPE pellets were kept at −80°C.

### RNA Isolation From Murine Eyecups

Mice were sacrificed and the eyes were enucleated. Adjacent tissues, such as muscle and fat, were removed and the cornea, lens, retina, and optic nerve were discarded. Remaining eyecups were homogenized in TRIzol Reagent (Invitrogen) using a pestle. For RNA extraction, TRIzol Reagent was used according to manufacturer's recommendations and RNA was stored at −80°C until usage.

### QuantSeq 3′ mRNA Sequencing Library Preparation

Preparation of libraries was performed with a total of 30 ng of RNA from each sample using QuantSeq 3′mRNA-Seq Library prep kit (Lexogen, Vienna, Austria) according to manufacturer's instructions. Total RNA was quantified using the Qubit 2.0 fluorimetric Assay (Thermo Fisher Scientific). Libraries were prepared from 30 ng of total RNA using the QuantSeq 3′ mRNA-Seq Library Prep Kit FWD for Illumina (Lexogen GmbH). Quality of libraries was assessed by using High Sensitivity DNA D1000 ScreenTape system (Agilent Technologies). Libraries were sequenced on a NovaSeq 6000 sequencing system using an S1, 100 cycles flow cell (Illumina Inc.). Amplified fragmented cDNA of 300 bp in size were sequenced in single-end mode with a read length of 100 bp. Illumina NovaSeq base call (BCL) files are converted in fastq file through bcl2fastq (version v2.20.0.422).

### QuantSeq 3′ mRNA Sequencing Data Processing and Analysis

For analysis, sequence reads were trimmed using BDduk software (https://jgi.doe.gov/data-and-tools/bbtools/bb-tools-user-guide/usage-guide/) (BBMap suite 37.31) to remove adapter sequences, poly-A tails and low-quality end bases (regions with average quality below 6). Alignment was performed with STAR 2.6.0a3 (Dobin et al., 2013) on mm10 reference assembly obtained from CellRanger website (https://support.10xgenomics.com/single-cell-gene-expression/software/release-notes/build#mm10_3.0.0; Ensembl assembly release 93). Expression levels of genes were determined with htseq-count (Anders et al., 2015) using Gencode/Ensembl gene model. All genes having <1 cpm in less than n_min samples and Perc MM reads > 20% simultaneously were filtered out. Differential expression analysis was performed using edgeR (Robinson et al., 2009), a statistical package based on generalized linear models, suitable for multifactorial experiments. A minimum of 3 biological replicates were used for statistics. The threshold for statistical significance chosen was False Discovery Rate (FDR) < 0.05: in detail, 592 genes were differentially expressed (290 genes induced and 302 inhibited) in the P11 dataset (GSE144845) while 2,276 genes were differentially expressed (1,056 genes induced and 1,220 inhibited) in the P29 dataset (GSE144846). Gene Ontology (GOEA) and Functional Annotation Clustering analyses were performed using DAVID Bioinformatic Resources (Huang et al., 2009a,b) restricting the output to Biological Process terms (BP_FAT). The threshold for statistical significance of GOEA was FDR < 0.1 and Enrichment score (ES) ≥1.5. The Enrichment score (ES) represents the amount to which genes in a gene ontology cluster are over-represented. We then compared the two datasets. The VENN diagram in Figure 1 summarizes the results after the comparison: we found 33 down-regulated and 32 up-regulated genes, respectively, in common in the two datasets genes and over 90 regulated in opposite correlation (44 down-regulated in P11 and up-regulated in P21; 55 up-regulated in P11 and down-regulated genes in P29).

### Data Visualization

Heatmaps (Figures 4B, 8B) were generated using custom annotated scripts.

### Quantitative Real-Time PCR (qPCR)

RNA was reverse transcribed to cDNA using GoTaq Probe 2-Step RT-qPCR System (Promega) and cDNA was stored at −20°C until usage. qPCR was performed via the StepOne-Plus Real-Time PCR System (Applied Biosystems) using Platinum SYBR Green (Invitrogen).

The following cycling conditions were used: 95°C for 10 min followed by 40 cycles of 95°C for 15 s, 60°C for 1 min. Relative target gene expression was normalized to *Tbp*. The primer sequences used are listed in Table 1. A minimum of 3 biological replicates were used for statistics.

**Table 1:** Sequences of used primers.

**Gene**	**Forward (5^**′**^-3^**′**^)**	**Reverse (5^**′**^-3^**′**^)**
Cdh1	ACTGTGAAGGGACGGTCAAC	GGAGCAGCAGGATCAGAATC
Cldn19	TCCTCTTGGCAGGTCTCTGT	GTGCAGCAGAGAAAGGAACC
Snail	TCCAAACCCACTCGGATGTGAAGA	TTGGTGCTTGTGGAGCAAGGACAT
Tbp	CTTCGTGCAAGAAATGCTGAAT	CAGTTGTCCGTGGCTCTCTTATT
Tjp1	GACCAATAGCTGATGTTGCCAGAG	TGCAGGCGAATAATGCCAGA

### Proteomics

For tissue lysis, RPE cells were transferred to 1.4 mm ceramic beads containing 0.5 ml Precellys® tubes and 100 μl lysis buffer per mg tissue was added. For lysis, samples were shaken in the Precellys® 24 system three times at 5,500 rpm for 20 s with 30 s of cooling on ice between each step. All lysates were incubated for 30 min at 4°C in an end-over-end shaker, centrifuged 10 min at 4°C with 10,000 g and supernatant was transferred to a new tube. The protein concentration was determined by a Bradford assay.

Affinity purified eluates were precipitated with chloroform and methanol followed by trypsin digestion as described before (Gloeckner et al., 2009). LC-MS/MS analysis was performed on Ultimate3000 RSLCnano systems (Thermo Scientific) coupled to an Orbitrap Fusion Tribrid mass spectrometer (Thermo Scientific) by a nano spray ion source. Tryptic peptide mixtures were injected automatically and loaded at a flow rate of 10 μl/min in 0.1% trifluoroacetic acid in HPLC-grade water onto a nano trap column (Thermo Scientific; Orbitrap Fusion: 2 mm x 10 mm, μPAC Trapping column, 300 nm, 100–200 Å, PharmaFluidics). After 3 min, peptides were eluted and separated on the analytical column (Orbitrap Fusion: 315 μm x 50 cm, μPACTM nano-LC columns-−50 cm μPACTM C18, 300 nm, 100–200 Å, PharmaFluidics) by a linear gradient from 2 to 30% of buffer B (80% acetonitrile and 0.08% formic acid in HPLC-grade water) in buffer A (2% acetonitrile and 0.1% formic acid in HPLC-grade water) at a flow rate of 300 nl/min over 95 min. Remaining peptides were eluted by a short gradient from 30 to 95% buffer B in 5 min. From the high-resolution MS pre-scan with a mass range of 335 to 1,500. The Orbitrap Fusion was run in top speed mode with a cycle time of 3 s. The normalized collision energy for HCD was set to a value of 30 and the resulting fragments were detected in the ion trap. The lock mass option was activated; the background signal with a mass of 445.12003 was used as lock mass (Olsen et al., 2005). Every ion selected for fragmentation was excluded for 20 s by dynamic exclusion.

MS/MS data were analyzed using the MaxQuant software (version 1.6.1.09) (Cox and Mann, 2008; Cox et al., 2009). As a digesting enzyme, Trypsin/P was selected with maximal 2 missed cleavages. Cysteine carbamidomethylation was set for fixed modifications, and oxidation of methionine and N-terminal acetylation were specified as variable modifications. The data were analyzed by label-free quantification (no fast LFQ) with the minimum ratio count of 2. The first search peptide tolerance was set to 20, the main search peptide tolerance to 4.5 ppm and the re-quantify option was selected. For peptide and protein identification, the following subset of the SwissProt database was used: mouse release 2019_08, #17,027 entries, contaminants were detected using the MaxQuant contaminant search. A minimum peptide number of 2 and a minimum length of 7 amino acids were tolerated. Unique and razor peptides were used for quantification. The match between run options was enabled with a match time window of 0.7 min and an alignment time window of 20 min. The statistical analysis was done using the Perseus software (version 1.6.2.3) (Tyanova et al., 2016). A minimum of 3 biological replicates were used for statistics. Potential contaminants, peptides only identified by side or reverse sequence were removed. Minimum half of the samples must have valid values. Based on the median value, significance A (Benjamini-Hochberg FDR) was calculated. The stability of protein ratios within groups was determined using the student's *t*-test. Only proteins with a significance A < 0.05 and a student's *t*-test *p* < 0.05 were taken as being significantly altered.

Gene enrichment analysis was performed using GetGo (http://getgo.russelllab.org/) (Boldt et al., 2016). Only gene names of proteins that showed a significance A < 0.05 and a student's *t*-test *p* < 0.05 for this analysis.

### Cell Morphology Assessment

After image acquisition the images were processed using a previously published trained neural network that can accurately identify cell borders in fluorescent images (Schaub et al., 2020). After the algorithm identified cell borders, the segmentation was manually validated for each image. If borders were incorrect, segmentations were manually corrected. Once validated, the images with the identified borders were then analyzed using the methodology outlined by Sharma et al. (2019). Cells in each image were assessed for area, perimeter, number of neighbors, elongation of the cell (Aspect ratio), how like regular convex polygons (Polygonality Score—Equations 1–3) cells were, how hexagonal (Hexagonality Score—Equations 4–6) cells were (Sharma et al., 2019), the standard deviation of the hexagonality of cells, Feret's Max diameter, Feret's minimum diameter, and the solidity of the cells.

(1) $$\begin{array}{l} PSR=\frac{P_{cell}}{P_{Hull}}\;*\;\left[1-|1-\frac{P_{Cell}}{N_{Neighbors}\sqrt{\frac{4\;*\;A_{Cell}}{N_{Neighbors}\;*\;\cot\frac{\pi }{N_{Neighbors}}}}}|\right] \end{array}$$

(2) $$\begin{array}{l} PAR=\;\frac{A_{Cell}}{A_{Hull}}\;*\;\left[1-|1-\frac{4\;*\;A_{Cell}}{N_{Neighbors}{\left(\frac{P_{Cell}}{N_{Neighbors}}\right)}^{2}\;*\;\cot\frac{\pi }{N_{Neighbors}}}|\right] \end{array}$$

(3) $$\begin{array}{l} Polygonality_{Score}=\;10\;*\;\left(\frac{PSR\;+PAR}{2}\right) \end{array}$$

(4) $$\begin{array}{l} HSR=\frac{P_{cell}}{P_{Hull}}\;*\;\left[1-|1-\frac{P_{Cell}}{6\;*\;\sqrt{\frac{4\;*\;A_{Cell}}{6\;*\;\cot\frac{\pi }{6}}}}|\right] \end{array}$$

(5) $$\begin{array}{l} HAR=\;\frac{A_{Cell}}{A_{Hull}}\;*\;\left[1-|1-\frac{4\;*\;A_{Cell}}{6\;*\;{\left(\frac{P_{Cell}}{6}\right)}^{2}\;*\;\cot\frac{\pi }{6}}|\right] \end{array}$$

(6) $$\begin{array}{l} Hexagonality_{Score}=10\;*\;\left(\frac{HSR\;+HAR}{2}\right) \end{array}$$

To calculate the above the python package SciPy 1.2.2 was used (Virtanen et al., 2020), with the addition of the polygonality score, hexagonality score, standard deviation of the hexagonality score, and number of neighbors. The calculations were validated on digitally created synthetic images of shapes and cells to ensure that the values that it produces were accurate.

All statistical analysis was performed using R (R. Foundation for Statistical Computing, 2017), the lme4 package (Bates et al., 2015), and the emmeans package (Searle et al., 1980). Data was first assessed for normality by determining data skewness, kurtosis, and q-q plots. All differences between *Bbs8*^+/+^ and *Bbs8*^−/−^ were assessed using linear mixed effects models controlling for repeated measures from each mouse. Linear marginal means rather than least squared means were used to determine the magnitude of differences. Differences in variance of the data was determined by taking the residual of all measures from their median and running the linear mixed effect model using residual values rather than raw data. A Bonferroni-Dunn correction was used to correct for the additional assessment of residuals, as well as raw data. All pair-wise comparisons were controlled for using Tukey's post test and an adjusted alpha of 0.05 was used for significance. All plots for shape/morphological measurements were done using the ggpllot2 package (Wickham, 2009).

### Retinal Adhesion Assay

For the retinal adhesion assay, we adapted a protocol previously described (Nandrot et al., 2006). Mice were sacrificed, eyes were enucleated and transferred into 1 × Hank's balanced salt solution containing Ca^2+^ and Mg^2+^ (HBSS+) (Gibco, #14025-092). Adjacent tissues, cornea and lens were removed. One eyecup at a time was transferred into a dry, empty dish and cut radially toward the optic nerve. The eyecup was then flattened, and the neural retina peeled off using forceps. Retinae were lysed individually in 50 mM Tris (pH 7.5), 2 mM EDTA, 150 mM NaCl, 1% Triton X-100, 0.1% SDS, and 1% NP-40, freshly supplemented with 1% protease and phosphatase inhibitors via sonication for 10 s on ice. After centrifugation (5 min, 21130 rcf, 4°C) lysates and pellets were kept separate on ice and protein content of the lysates were quantified using bicinchoninic acid (BCA) assay. For melanin dissolution, the pellets were washed in 100 μl 50% ethanol and 50% diethylether (10 min, 21130 rcf). Supernatant was discarded, pellets were dissolved in 150 μl 20% DMSO, 2 M NaOH and incubated for 30 min at 60°C. To quantify melanin concentration, absorbance of samples and commercially available melanin (Sigma-Aldrich, #M0418) dissolved in 20% DMSO, 2 M NaOH at defined concentrations were measured at 490 nm. Individual melanin concentrations were normalized to the corresponding protein concentration to calculate the concentration of melanin per milligram of protein.

### Cell Culture

ARPE-19 cells were kindly provided from Karsten Boldt (Tübingen) and cultured in DMEM/F-12 + GlutaMax (Gibco, #31331028) supplemented with 10% fetal bovine serum (FBS) (LONZA), and 1% penicillin/streptomycin (Thermo Fischer, #10378016) (referred to as complete medium). *BBS8* siRNA (Dharmacon, #L-021417-02-0005) and non-targeting siRNA (IDT, TriFECTa) were used to generate *BBS8 KD* and *Non-targeting control (NTC)* cells. Transfections were performed using antibiotic-free medium in 96-well plates using Lipofectamine RNAiMAX reagent (Invitrogen, #113778150) according to manufacturer's recommendations for reverse transfections. Post transfection, the cells we cultured in complete medium. The phagocytosis assay was performed 48 h post-transfection.

### Phagocytosis Assay

Isolation and preparation of bovine POS were described previously (Nandrot et al., 2007). For phagocytosis assay, we adapted a protocol previously described (Nandrot et al., 2007). Confluent *BBS8 KD* and *NTC* cells were preincubated for 1 h in serum- and antibiotic-free DMEM/F-12 + GlutaMax followed by incubation with POS for 7 h at 37°C in the dark. To remove excess POS, three washing steps with PBS supplemented with 0.2 mM Ca^2+^ and 1 mM Mg^2+^ (PBS-CM) were performed. In order to differentiate between bound and internalized POS, parallel wells were incubated with 4% trypan blue for 10 min followed by two washing steps with PBS-CM. Addition of trypan blue quenches surface fluorescence and therefore the two populations (internalized vs. bound) can be distinguished (bound = total – internalized). All wells were fixed in ice-cold methanol for 10 min, rehydrated in PBS-CM for 10 min at room temperature and incubated with DAPI (1:400) for 15 min. Following this, two washing steps with PBS-CM were performed and an equal volume of PBS-CM was added to all wells. FITC-POS and DAPI-labeled cells were quantified using the Infinite M1000 Pro microplate reader and associated Magellan software (Tecan).

### Statistics

Unless indicated differently, statistical analysis was performed using GraphPad Prism 6.0. ROUT test (Q = 0.1%) was performed prior to statistical analysis. Used statistical tests are stated in corresponding figure legends. For qPCR data a one sample *t*-test with a theoretical mean set as 1 (WT = *Bbs8*^+/+^ value) was performed. For the retinal adhesion analysis, a D'Agostino & Pearson omnibus normality test was performed to check that the results are normally distributed. Following this, an unpaired *t*-test was performed. For the phagocytosis assay, a one-way ANOVA and Tukey *post-hoc* test was performed. Images and data were analyzed blinded to genotype. See sections Cell morphology assessment, QuantSeq 3′ mRNA sequencing data processing and analysis and Proteomics for more details. Sample size was determined by availability of tissue with a minimum of three biological replicates.

## Data Availability Statement

The datasets presented in this study can be found in online repositories. The names of the repository/repositories and accession number(s) can be found in the article/Supplementary Material.

## Ethics Statement

The animal study was reviewed and approved by the Landesuntersuchungsamt Rheinland-Pfalz and were performed in accordance with the guidelines given by the ARVO Statement for the Use of Animals in Ophthalmic and Vision Research. Animal maintenance and handling were performed in line with the Federation for Laboratory Animal Science Associations (FELASA) recommendations.

## Author Contributions

SS, RD, NH, KB, IC, and HLM-S designed experiments. SS, RD, JN, VK, PAM DI, NH, and KB performed experiments and analyzed data. SS and HLM-S wrote the paper, and conceived and directed the study. All authors revised the manuscript.

## Conflict of Interest

The authors declare that the research was conducted in the absence of any commercial or financial relationships that could be construed as a potential conflict of interest.

## Supplementary Material

Supplementary Figure 1 *Bbs8*-deficient RPE show changes in cell morphological parameters**. (A–F)** Quantification of cell morphology parameters. Statistical analysis is described in methods. Comparison of **(A)** eccentricity, **(B)** minFeret, **(C)** maxFeret, **(D)** solidity, **(E)** equivalent diameter, and **(F)** number of neighbors of *Bbs8*^−/−^ and *Bbs8*^+/+^ RPE cells show no significant changes (*n*: P0 = 6 eyes, P11 = 9 eyes, P29 = 4 eyes, P81 = 3 eyes). Significance levels: mean: > 0.05 not significant (ns), ≤ 0.05^*^, ≤ 0.01^**^, ≤ 0.001^***^; variance: > 0.05 not significant (ns), ≤ 0.05#, ≤ 0.01##, ≤ 0.001###. **(G)** Representative images of RPE flatmounts stained for ZO-1 (green) to visualize the cell membrane and DAPI for nuclear DNA. In *Bbs8*^−/−^ RPE we identified discontinuous membrane staining at all ages. These areas were excluded from cell morphology assessment. Scale bar: 25 μm.

Supplementary Figure 2 Deletion of *Bbs8* leads to changes in apical microvilli. Representative images of RPE flatmounts (Z-stack) stained for p-ERM (orange) and F-actin (red) to visualize apical microvilli and the cytoskeleton. DAPI was used to stain nuclear DNA. P11 *Bbs8*^−/−^ RPE shows abnormal accumulations of p-ERM staining (arrowheads). Images were taken using a Confocal Zeiss LSM 9 with Airyscan 9. Scale bars: 10 μm.

Supplementary Figure 3 Optimization of phagocytosis assays using ARPE-19 cells. **(A)** Quantification of *BBS8* expression after knockdown using *BBS8* siRNA from Dharmacon (dh) and IDT compared to non-targeting control (NTC) (*n* = 3). Due to higher efficacy of the siRNA from Dharmacon, further experiments were performed using this. Statistical analysis was performed using one-way ANOVA and Dunnett's test *post-hoc* test. Significance levels: > 0.05 not significant (ns), ≤ 0.05^*^, ≤ 0.01^**^, ≤ 0.001^***^. **(B)** Comparison of total, bound and internalized fluorescently labeled photoreceptor outer segments (POS). FITC/DAPI ratio measured after incubation of ARPE19 cells for 5 and 7 h, respectively.

Supplementary Figure 4 Deletion of *Bbs8* leads to increased expression of Snail and changes ciliation in the RPE. **(A)** Representative images of P11 RPE flatmounts stained for Snail (red) and ZO-1 (green). DAPI was used to stain nuclear DNA. P11 *Bbs8*^−/−^ RPE shows an increase in Snail expression in the nuclear region, particularly in cells with disrupted morphology. Scale bars: 25 μm. **(B)** Representative images of P81 RPE flatmounts (Z-stack) stained for primary cilia (arrowheads) using Arl13b (red) and Gt335 (green). F-Actin (magenta) was stained to visualize the cytoskeleton and DAPI was used to stain nuclear DNA. Co localization of Arl13b and transition zone marker Gt335 is required to determine a true primary cilium, which is more readily identified in the mutant (white arrows). Scale bars: 10 μm. Images were taken using a Leica DM6000B microscope.

Supplementary Table 1 Differentially expressed genes (DEGs) significantly regulated at P11. Significance defined by using False Discovery Rate (FDR) < 0.05 as threshold. 290 genes induced in red, and 302 inhibited in green.

Supplementary Table 2 Differentially expressed genes (DEGs) significantly regulated at P29. Significance defined by using False Discovery Rate (FDR) < 0.05 as threshold. 1,056 genes induced in red; and 1220 inhibited in green.

Supplementary Table 3 Comparison of differentially expressed genes (DEGs) between Bbs8^−/−^ and control with RPE-specific gene lists.

Supplementary Table 4 Gene Ontology enrichment analysis of the differentially expressed genes (DEGs) between Bbs8^−/−^ and control at P11. The Biological Process (BP_FAT) terms enriched among the down-regulated (*n* = 302) and up-regulated (*n* = 290) genes are highlighted in green (A) and red (B), respectively. For each BP annotation cluster, the GO identifier (“GO:”), associated term (“Term”) and Enrichment score are provided. The genes belonging to each term are listed in column D (and their total number in column C under “count”). False Discovery Rate score (FDR) is shown in column E.

Supplementary Table 5 Gene Ontology enrichment analysis of the differentially expressed genes (DEGs) between Bbs8-/- and control at P29. The Biological Process (BP_FAT) terms enriched among the down-regulated (*n* = 1,220) and up-regulated (*n* = 1,056) genes are highlighted in green (A) and red (B), respectively. For each BP annotation cluster, the GO identifier (“GO:”), associated term (“Term”) and Enrichment score are provided. The genes belonging to each term are listed in column D (and their total number in column C under “count”). False Discovery Rate score (FDR) is shown in column E.

Supplementary Table 6 Gene Ontology enrichment analysis of the differentially expressed genes (DEGs) resulting from the comparison of the two datasets at P11 and P29. The Biological Process (BP_FAT) terms enriched among the down-regulated genes in both datasets (*n* = 33) and up-regulated in both datasets (*n* = 32) genes are highlighted in green (A) and red (B), respectively. For each BP annotation cluster, the GO identifier (“GO:”), associated term (“Term”) and Enrichment score are provided. The genes belonging to each term are listed in column D (and their total number in column C under “count”). False Discovery Rate score (FDR) is shown in column E.
