# Supplementary figures and images for: Loss of Ciliary Gene Bbs8 Results in Physiological Defects in the Retinal Pigment Epithelium
Source: Front Cell Dev Biol. 2021 Feb 18;9:607121. doi: 10.3389/fcell.2021.607121 (PMC7930748; doi:10.3389/fcell.2021.607121)

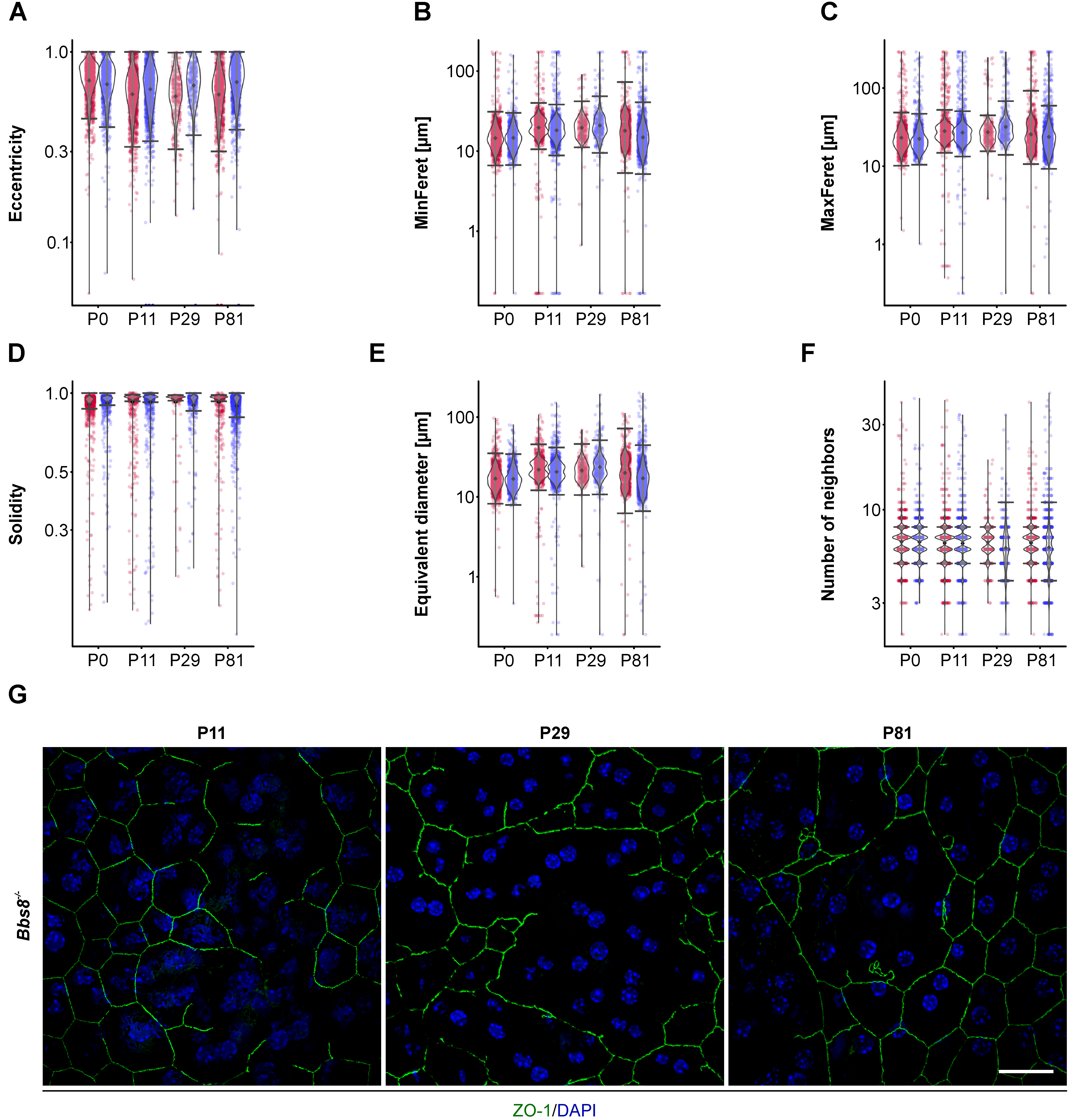

Supplement: Supplementary Figure 1 — Bbs8-deficient RPE show changes in cell morphological parameters. (A–F) Quantification of cell morphology parameters. Statistical analysis is described in methods. Comparison of (A) eccentricity, (B) minFeret, (C) maxFeret, (D) solidity, (E) equivalent diameter, and (F) number of neighbors of Bbs8−/− and Bbs8+/+ RPE cells show no significant changes (n: P0 = 6 eyes, P11 = 9 eyes, P29 = 4 eyes, P81 = 3 eyes). Significance levels: mean: > 0.05 not significant (ns), ≤ 0.05*, ≤ 0.01**, ≤ 0.001***; variance: > 0.05 not significant (ns), ≤ 0.05#, ≤ 0.01##, ≤ 0.001###. (G) Representative images of RPE flatmounts stained for ZO-1 (green) to visualize the cell membrane and DAPI for nuclear DNA. In Bbs8−/− RPE we identified discontinuous membrane staining at all ages. These areas were excluded from cell morphology assessment. Scale bar: 25 μm. [file Image_1.TIF]

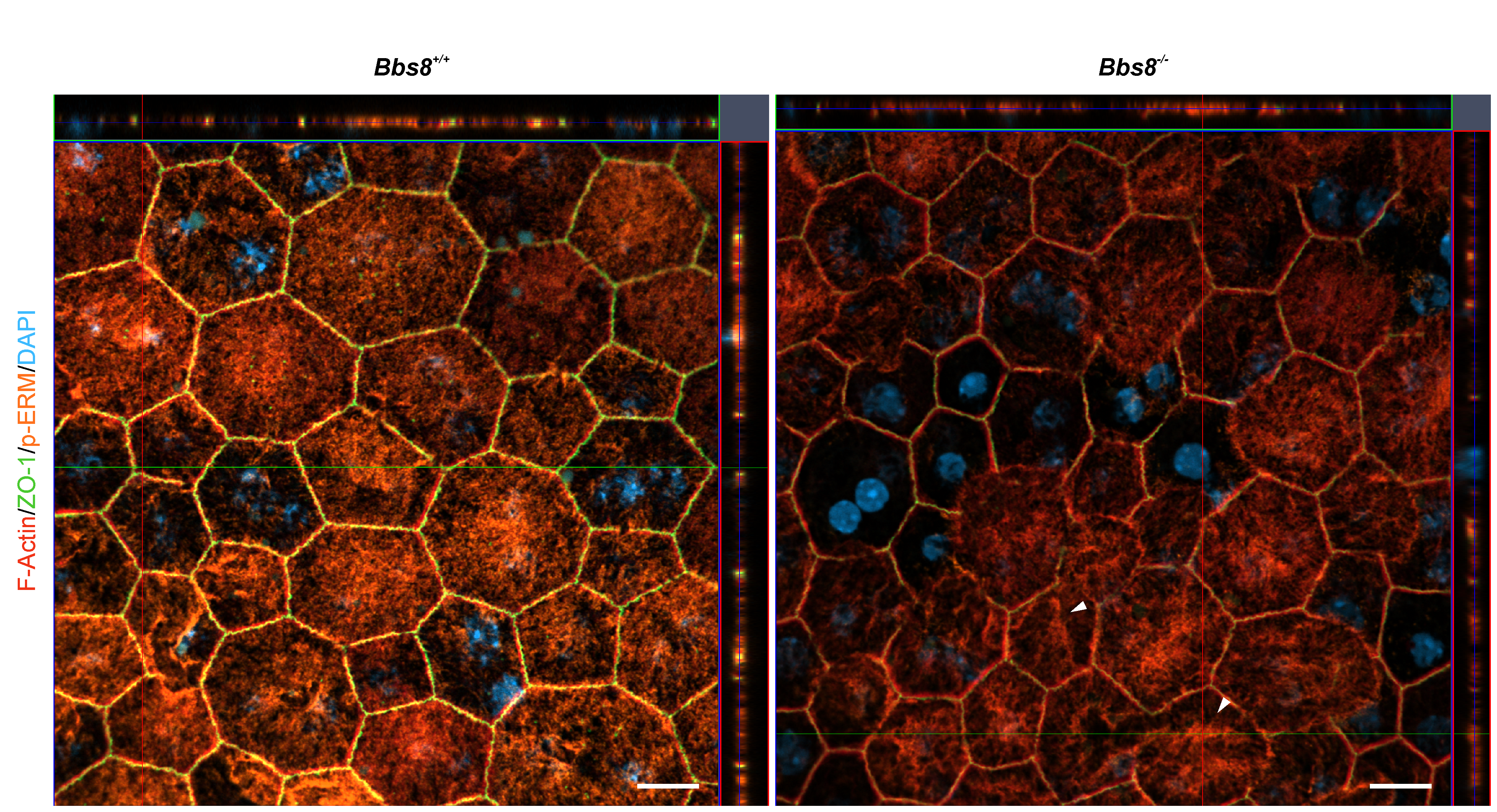

Supplement: Supplementary Figure 2 — Deletion of Bbs8 leads to changes in apical microvilli. Representative images of RPE flatmounts (Z-stack) stained for p-ERM (orange) and F-actin (red) to visualize apical microvilli and the cytoskeleton. DAPI was used to stain nuclear DNA. P11 Bbs8−/− RPE shows abnormal accumulations of p-ERM staining (arrowheads). Images were taken using a Confocal Zeiss LSM 9 with Airyscan 9. Scale bars: 10 μm. [file Image_2.TIF]

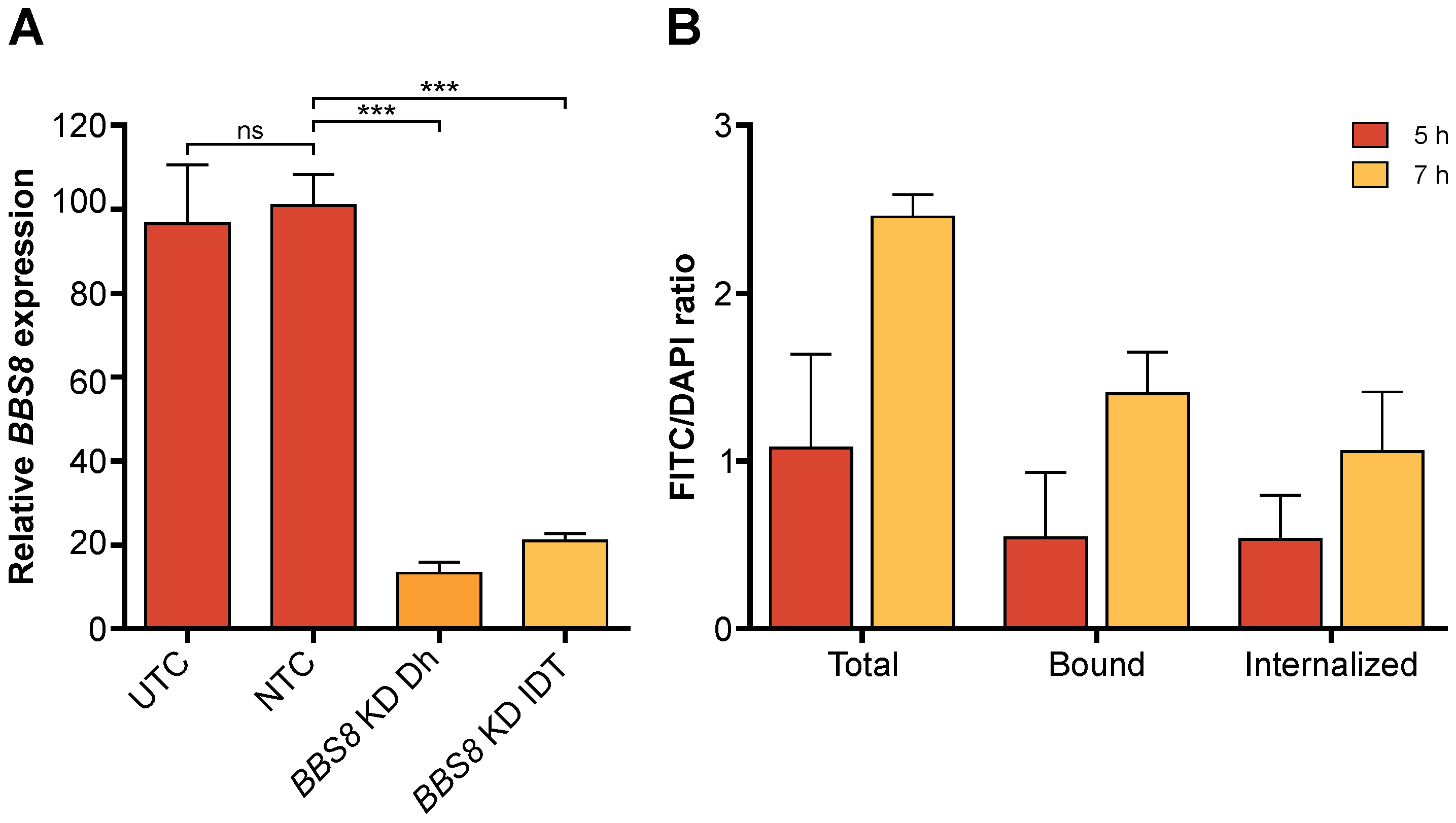

Supplement: Supplementary Figure 3 — Optimization of phagocytosis assays using ARPE-19 cells. (A) Quantification of BBS8 expression after knockdown using BBS8 siRNA from Dharmacon (dh) and IDT compared to non-targeting control (NTC) (n = 3). Due to higher efficacy of the siRNA from Dharmacon, further experiments were performed using this. Statistical analysis was performed using one-way ANOVA and Dunnett's test post-hoc test. Significance levels: > 0.05 not significant (ns), ≤ 0.05*, ≤ 0.01**, ≤ 0.001***. (B) Comparison of total, bound and internalized fluorescently labeled photoreceptor outer segments (POS). FITC/DAPI ratio measured after incubation of ARPE19 cells for 5 and 7 h, respectively. [file Image_3.TIF]

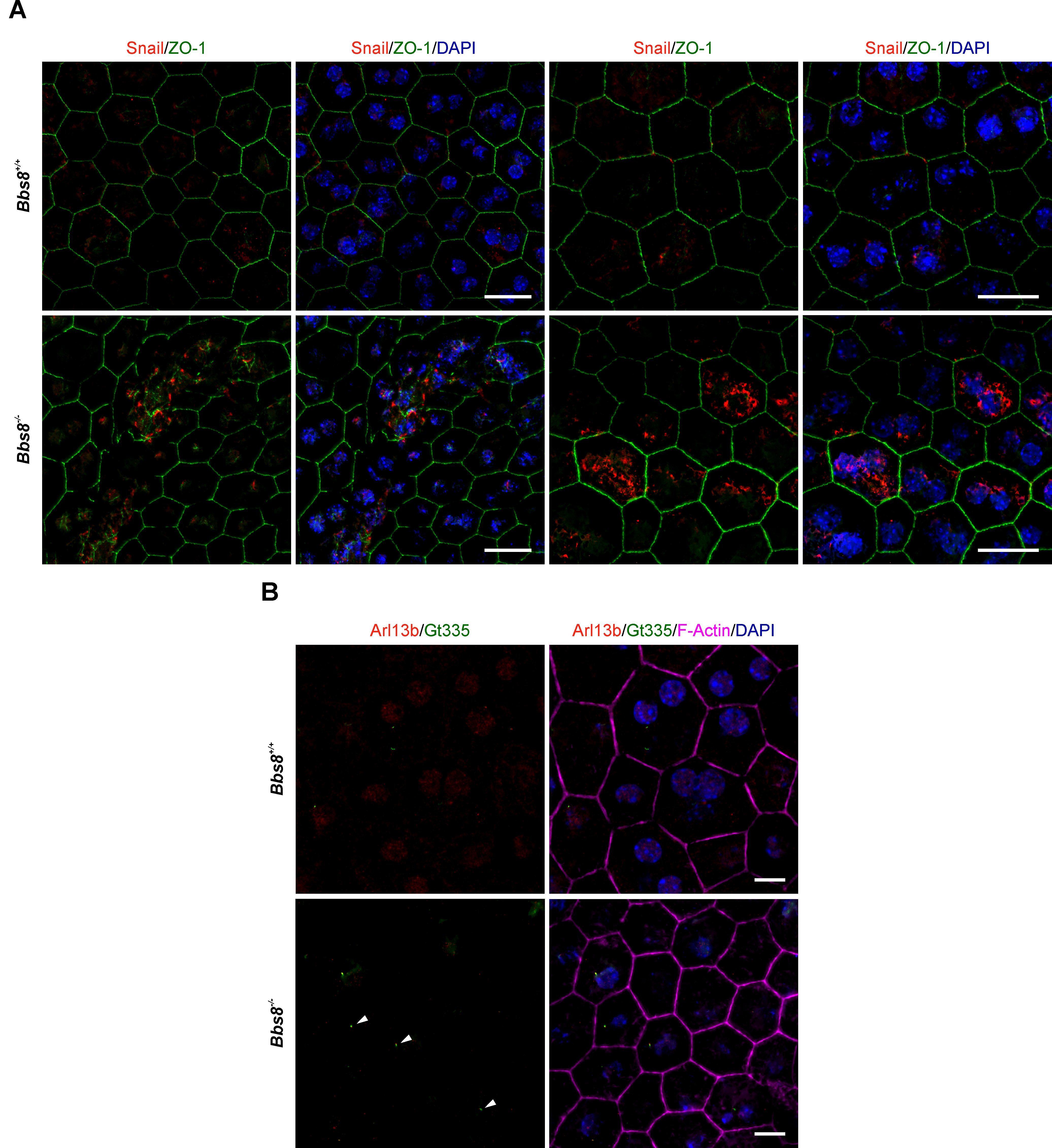

Supplement: Supplementary Figure 4 — Deletion of Bbs8 leads to increased expression of Snail and changes ciliation in the RPE. (A) Representative images of P11 RPE flatmounts stained for Snail (red) and ZO-1 (green). DAPI was used to stain nuclear DNA. P11 Bbs8−/− RPE shows an increase in Snail expression in the nuclear region, particularly in cells with disrupted morphology. Scale bars: 25 μm. (B) Representative images of P81 RPE flatmounts (Z-stack) stained for primary cilia (arrowheads) using Arl13b (red) and Gt335 (green). F-Actin (magenta) was stained to visualize the cytoskeleton and DAPI was used to stain nuclear DNA. Co localization of Arl13b and transition zone marker Gt335 is required to determine a true primary cilium, which is more readily identified in the mutant (white arrows). Scale bars: 10 μm. Images were taken using a Leica DM6000B microscope. [file Image_4.TIF]
